# LncRNA-induced lysosomal localization of NHE1 promotes increased lysosomal pH in macrophages leading to atherosclerosis

**DOI:** 10.1016/j.jbc.2025.110246

**Published:** 2025-05-16

**Authors:** Pengcheng Shi, Bo Tang, Wen Xie, Ke Li, Di Guo, Yining Li, Yufeng Yao, Xiang Cheng, Chengqi Xu, Qing K. Wang

**Affiliations:** 1 Center for Human Genome Research, Key Laboratory of Molecular Biophysics of the Ministry of Education, College of Life Science and Technology, Wuhan, P. R. China; 2 Department of Cardiology, Union Hospital, Tongji Medical College, Wuhan, P. R. China; 3 Maternal and Child Health Hospital of Hubei Province, Women and Children's Hospital of Hubei Province, Huazhong University of Science and Technology, Wuhan, P. R. China

**Keywords:** *ANRIL*, *miR-181b-5p*, TMEM106B, NHE1, lysosomal pH, macrophage, atherosclerosis, coronary artery disease

## Abstract

*ANRIL*, also referred to as *CDKN2B-AS1*, is an lncRNA gene implicated in the pathogenesis of multiple human diseases including atherosclerotic coronary artery disease; however, definitive *in vivo* evidence is lacking and the underlying molecular mechanism is largely unknown. In this study, we show that *ANRIL* overexpression causes atherosclerosis *in vivo* as transgenic mouse overexpression of full-length *ANRIL* (NR_003529) increases inflammation and aggravates atherosclerosis under *ApoE*^*−/−*^ background (*ApoE*^*−/−*^*ANRIL* mice). Mechanistically, *ANRIL* reduces the expression of *miR-181b-5p*, which leads to increased TMEM106B expression. TMEM106B is significantly upregulated in the atherosclerotic lesions of both human CAD patients and *ApoE*^*−/−*^*ANRIL* mice. TMEM106B interacts and colocalizes with Na^+^-H^+^ exchanger NHE1, which results in the mislocalization of NHE1 from cell membranes to lysosomal membranes, leading to increased lysosomal pH in macrophages. Large truncation and point mutation analyses define the critical amino acids for TMEM106B–NHE1 interaction and lysosomal pH regulation as F115 and F117 on TMEM106B and I537, C538, and G539 on NHE1. Topological analysis suggests that both N terminus and C terminus of NHE1 are located inside lysosomal lumen, consistent with our finding that NHE1 is an important new proton efflux channel involved in raising lysosomal pH. A short TMEM106B peptide (YGRKKRRQRRR-L_111_A_112_V_113_F_114_F_115_L_116_F_117_) disrupting the TMEM106B–NHE1 interaction normalized lysosomal pH in macrophages with *ANRIL* overexpression. Our data demonstrate that *ANRIL* promotes atherosclerosis *in vivo* and identify the *ANRIL*–*miR-181b-5p*–TMEM106B-NHE1–lysosomal pH axis as the underlying molecular pathogenic mechanism for the chromosome 9p21.3 genetic locus for coronary artery disease.

Coronary artery disease (CAD) is the leading cause of death worldwide and is caused by atherosclerotic plaques built in coronary arteries (1, 2, 3). In 2007, a series of large-scale genome-wide association studies (GWAS) identified the most robust risk locus for CAD on chromosome 9p21.3 (4, 5, 6). Since then, GWAS have identified >200 risk loci for CAD and its major complication, myocardial infarction (MI). However, it has been highly challenging to elucidate how each locus exerts its genetic effect, define the specific disease-causing gene(s), and determine the underlying molecular mechanisms. The 9p21.3 CAD locus shows the strongest effect on CAD risk and explains 13% of CAD incidences, making it the most important genetic locus for CAD to date (7). This locus contains a 60-kb linkage disequilibrium (LD) block with a large cluster of 59 to 100 single nucleotide polymorphisms that was associated with a risk of CAD (6).

The 9p21.3 CAD locus contains multiple genes, including *MTAP* encoding 5′-methylthioadenosine phosphorylase, *CDKN2A* encoding p16, *CDKN2B* encoding p15, and *ANRIL* encoding a long noncoding RNA. Some studies suggested that *CDKN2A/B* was the causative gene, while other studies showed that *CDKN2A* and *CDKN2B* had no effect on the development of coronary atherosclerosis (8, 9, 10, 11). Some studies, including our studies, suggested that *ANRIL* was the causative gene. First, the 60-kb CAD-associated LD block is located at the 3′end of *ANRIL*, far away from *CDKN2A* and *CDKN2B* (12). Second, our previous study showed that one short transcript of *ANRIL*, DQ485454, was significantly downregulated in coronary arterial tissue samples from CAD patients compared with normal samples, whereas the longest *ANRIL* transcript (full-length NR_003529) was upregulated in CAD coronary artery samples (13). Further studies showed that the DQ485454 transcript was the major *ANRIL* transcript in endothelial cells (ECs) localized into the nucleus and significantly impaired two critical cellular processes involved in the initiation of atherosclerosis, including the monocyte adhesion to EC layer and transendothelial monocyte migration (14). The full-length NR_003529 *ANRIL* transcript showed the opposite effects in ECs (13). Consistent with our findings, Jarinova *et al.* showed that the expression of NR_003529 was increased, but the expression of shorter variants DQ485454 and EU741058 was decreased in homozygous carriers with 9p21.3 CAD risk alleles (15). Another study also showed significantly increased expression levels of NR_003529 and EU741058, but not DQ485454, in patients with a high atherosclerotic plaque load (16). However, a separate study showed that the atherosclerosis risk allele was associated with the reduced transcription of all four INK4/ARF-related transcripts, including *ANRIL* (17). In addition to linear *ANRIL*, circular *ANRIL* (*CircANRIL*) was also detected (18), and the predominant *CircANRIL* consisted of exons 5, 6, and 7 (19). Higher expression of *CircANRIL* was detected in individuals carrying the CAD-protective haplotype at 9p21.3 (19). CAD patients with >50% stenosis showed significantly less *CircANRIL* expression in peripheral blood mononuclear cells (PBMC) than CAD patients with <50% stenosis or non-CAD subjects (19). However, these correlation studies on *ANRIL* expression could not distinguish the cause-consequence effect of the expression changes on CAD. It is paramount to further characterize the role and mechanism of *ANRIL* in atherosclerosis, in particular, using transgenic mouse models.

Atherosclerosis is a chronic multifactorial vascular disease driven by the accumulation of lipids in the inner wall of arteries and lipid-driven inflammation, which leads to the narrowing and blockage of blood vessels, leading to CAD, stroke, and MI (20). Accumulation of lipids, mainly low-density lipoprotein (LDL) cholesterol, leads to initial intimal damage and inflammation, which attracts circulating monocytes to the damaged endothelial area (21). Monocytes are then transported to the intima of the artery by transendothelial monocyte migration and converted into macrophages, which are further transformed into foam cells (early atherosclerotic lesions) by ingesting large amounts of LDL cholesterol and other materials (22). The aggregation of foam cells forms lesion areas and contributes to lipid storage and plaque growth. The formation of the lesion triggers signals that attract vascular smooth muscle cells (VSMCs) to the injured area, which then begin to proliferate and produce extracellular matrix, eventually leading to atherosclerotic plaques (22). *ANRIL* was previously studied in detail in ECs and VSMCs; however, its role in macrophages remains unclear.

*TMEM106B* encodes a transmembrane protein 106 with 274 amino acids and a type II single transmembrane region (23). TMEM106B is localized on the surface of lysosomes with the N terminus extended into the cytoplasm (24). GWAS found that TMEM106B variants were associated with the risk of frontotemporal lobar degeneration (25), Alzheimer's disease (26), and Parkinson's disease (27). A cryo-EM structural study showed that C-terminal residues 120 to 254 of TMEM106B formed amyloid fibrils like TDP43 in human brains and correlated with age (28). However, the function of the N-terminal residues 1 to 119 of TMEM106B is not yet known. In neurodegenerative diseases, overexpression of TMEM106B led to impaired lysosomal acidification, abnormal degradation of endocytic cargos, and transport of lysosomes between neurons (29, 30). Recently, our group reported that *TMEM106B* variants were significantly associated with the risk of CAD and showed significant gene–gene interaction with *ANRIL* variant rs2383207 (31). Moreover, both *ANRIL* (NR_003529) and *TMEM106B* were upregulated in coronary artery tissues from patients with CAD (13, 31). However, the molecular mechanism by which TMEM106B upregulation causes CAD is unknown.

NHE1 is a Na^+^-H^+^ exchanger that regulates intracellular pH by squeezing out a proton in exchange for an extracellular Na^+^, thereby protecting cells from internal acidification (32). NHE1 activity was shown to be associated with the apoptosis of cardiomyocytes, fibroblasts, and macrophages by regulating intracellular pH (33). *ApoE*^*−/−*^*Nhe1*^*+/−*^ mice decreased atherosclerosis by reducing extracellular acidity of plaques, suggesting that NHE1 plays a role in the regulation of atherosclerosis (33, 34).

The pH in lysosomes is different from the cytoplasmic pH of 7.2 and maintained at an optimal and highly acidic pH of 4.5 to 5.0 for effective clearance of unwanted intracellular and extracellular components, cell debris, and toxic protein aggregates (35). The homeostasis of lysosomal pH is expected to be crucial for cellular homeostasis, as lysosomes are catabolic organelles that act as recycling hubs for numerous intracellular and extracellular components. The acidic lysosomal pH is maintained by V-ATPase, the vacuolar H^+^ ATPase that uses ATP as metabolic energy to pump protons into the lysosome lumen, and in exchange, CLC-7, a Cl^-^/H^+^ antiporter, moves Cl^-^ into lysosomes (35, 36). However, the role of CLC7 in the counter-H^+^ pathway was questioned, and an alternative of both Na^+^ and K^+^ as the counter ions was proposed (37). *TMEM175*, a susceptibility gene identified for Parkinson disease by GWAS, encodes a lysosomal potassium channel under neutral pH, but recent studies suggest that TMEM175 is also an H^+^-activated H^+^ channel under a hyperacidified condition as in lysosomes (38). As a lysosomal membrane protein, TMEM106B has been shown to be involved in the regulation of lysosomal pH (23, 39, 40, 41). However, the mechanism by which TMEM106B mediates the regulation of lysosomal pH and whether there are other channels on the lysosomal membrane involved in proton transport are unknown. Moreover, it is unknown about the functional role of abnormal lysosomal pH homeostasis in atherosclerosis.

The *ApoE* gene encodes Apolipoprotein E (APOE), which is involved in the regulation of lipoprotein metabolism and lipoprotein-mediated lipid transport (42). APOE mediates the binding of lipoproteins or lipid complexes in the plasma or interstitial fluids to specific cell surface receptors such as LDL receptor and other LDL receptor–related proteins to promote receptor-mediated endocytosis of very low-density lipoprotein remnants and chylomicrons (42). *ApoE*-deficient mice showed impaired clearance of very low-density lipoprotein remnants and chylomicrons (42, 43). After treatment with a high fat diet, *ApoE*^*−/−*^ mice developed dyslipoproteinemia, hypercholesterolemia, and atherosclerotic lesions rapidly (42, 43). *ApoE*^*−/−*^ mice are the ideal model for the study of atherosclerosis and also one of the most used mouse models in the field. Atherosclerosis is difficult to be detected for most genetically engineered mice, and therefore, these mice are often crossed to *ApoE*^*−/−*^ mice to make the detection of the atherosclerotic phenotypes easier (42, 43, 44). To identify the *in vivo* role and mechanism of *ANRIL* in atherosclerosis, we developed transgenic mice with the overexpression of *ANRIL* (full-length NR_003529) and crossed these mice to *ApoE*^*−/−*^ mice, resulting in *ApoE*^*−/−*^*ANRIL* mice. *ApoE*^*−/−*^*ANRIL* mice developed inflammation and atherosclerosis, providing the first *in vivo* evidence to strongly support that *ANRIL* is the atherosclerotic CAD gene at the 9p21.3 CAD locus. Further characterization of *ApoE*^*−/−*^*ANRIL* mice and human atherosclerotic tissue samples identified the *ANRIL*–*miR-181b-5p*–TMEM106B-NHE1–lysosomal pH axis as the underlying molecular pathogenic mechanism for CAD. Biochemical characterization showed that TMEM106B–NHE1 interaction was involved in the regulation of lysosomal pH, implicating that similar to TMEM175, NHE1 is a novel H^+^ efflux channel of lysosomes.

## Results

### *ANRIL* overexpression causes atherosclerosis in mice

**Figure 1:**
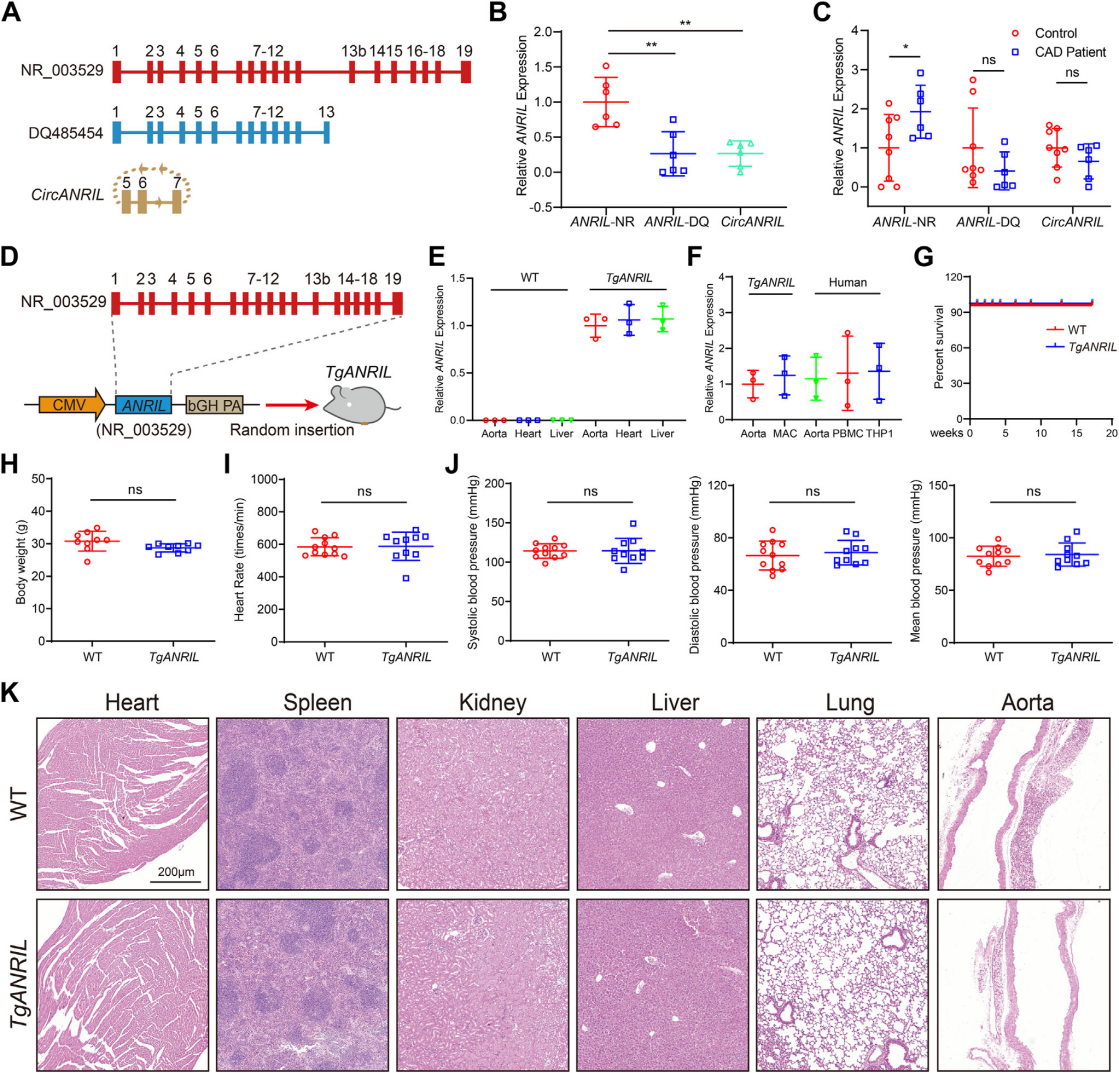
***ANRIL* expression was increased in human CAD patients and the development of *TgANRIL* mice.***A*, diagram showing the three major transcripts of *ANRIL*. *B*, RT-qPCR analysis to examine expression of different *ANRIL* transcripts in human CAD patients. n = 6. *C*, RT-qPCR analysis showed that the expression of *ANRIL* NR_003529 was upregulated in human CAD patients compared with controls. n = 6 to 8. *D*, transgenic construct pcDNA3.1-*ANRIL* (NR_003529) for the development of *TgANRIL* mice. *E*, RT-qPCR analysis was used to detect *ANRIL* (NR_003529) expression in tissues from WT mice and *TgANRIL* mice. n = 3. *F*, RT-qPCR analysis was used to detect *ANRIL* (NR_003529) expression in *TgANRIL* mice and humans. n = 3. *G*, survival rate of WT and *TgANRIL* mice. n = 12. *H*, body weight of WT and *TgANRIL* mice. n = 9. *I*, heart rate of WT and *TgANRIL* mice. n = 10 to 11. *J*, systolic blood pressure, diastolic blood pressure, and mean blood pressure of WT and *TgANRIL* mice. n = 10 to 11. *K*, HE staining of major organs, including the heart, spleen, kidney, liver, lung, and aorta. n = 3. ∗*p* < 0.05, ∗∗*p* < 0.01, ns, not significant. *B*, one-way ANOVA; (*C*) multiple *t* test; (*H*–*J*) unpaired two-tailed *t* test.

We investigated the three main transcripts of *ANRIL* (Fig. 1*A*) for their expression levels in human CAD arterial tissue samples and found that the expression of full-length *ANRIL* (NR_003529) was the highest (Fig. 1*B*). We also found that NR_003529 was significantly upregulated in CAD aortic tissue samples (Fig. 1*C*). Thus, we focused on NR_003529 for further studies and generated transgenic mice with the overexpression of NR_003529, referred to as *TgANRIL* (Fig. 1*D*). RT-qPCR analysis showed that *ANRIL* (NR_003529) was successfully expressed in the aorta, heart, and liver of *TgANRIL* mice (Fig. 1*E*). Importantly, the expression level of *ANRIL* in *TgANRIL* mice was comparable to that in human aorta, PBMCs, and THP1 monocytes (Fig. 1*F*). Thus, the basic pattern of *ANRIL* expression in *TgANRIL* mice reflects that of human cells and tissues. Compared with WT mice, *TgANRIL* mice did not show significant differences in the survival rate, body weight, heart rate, blood pressure, and gross anatomical structures of the heart, spleen, kidney, liver, lung, and aorta at the baseline (Fig. 1, *G–K*).

**Figure 2:**
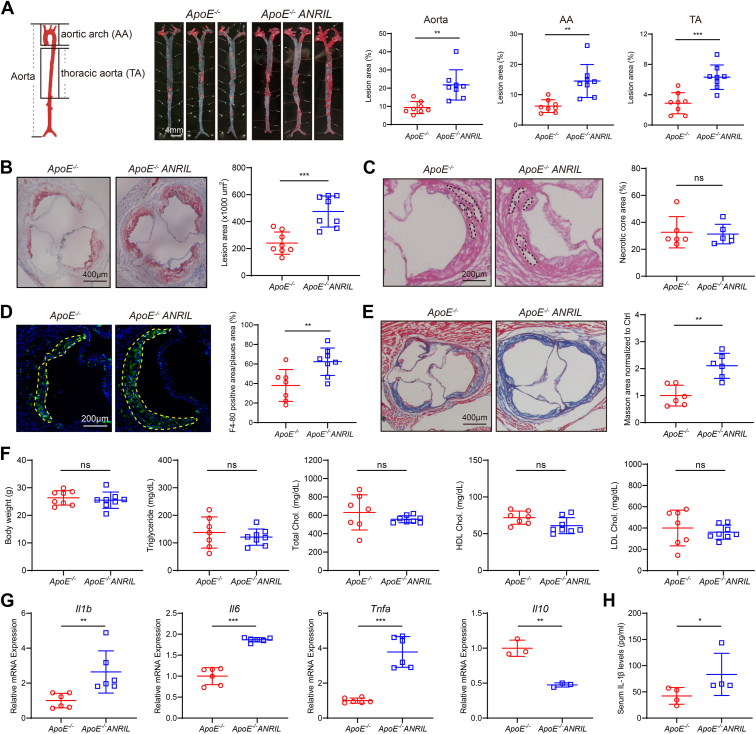
**Male *ApoE*^*−/−*^*ANRIL* mice show aggravated atherosclerosis.***A*, male mice were fed with a standard chow diet for 4 weeks and then with a WD for 14 weeks. Representative lipid/oil red O–stained enface images of aortas. n = 8. *B*, representative lipid/oil red O staining of aortic root sections. n = 8. *C*, representative HE staining of aortic root sections. n = 6. *D*, representative anti-F4-80 immunofluorescent images of aortic root sections. n = 7 to 8. *E*, representative Masson staining of aortic root sections. n = 6. *F*, body weight, TG, TC, HDL-c, and LDL-c levels. n = 7 to 8. *G*, RT-qPCR analysis for *Il1b*, *Il6*, *Tnfa*, and *Il10*. n = 3 to 6. *H*, ELISA to measure secreted serum IL-1β. n = 4. ∗*p* < 0.05, ∗∗*p* < 0.01, ∗∗∗*p* < 0.001, ns, not significant. *A*, *B*, *D*–*F*, and *G*, (*Il6*, *Il10*, and *Tnfa*), unpaired two-tailed *t* test; (*C* and *G*) (*Il1b*), and (*H*) nonparametric test.

To study the effect of *ANRIL* on atherosclerosis in *TgANRIL* mice *in vivo*, we crossed *TgANRIL* mice with *ApoE*^*−/−*^ mice to develop *ApoE*^*−/−*^*ANRIL* mice and assessed aortic atherosclerosis after feeding with a Western diet for 14 weeks. Male *ApoE*^*−/−*^*ANRIL* mice developed more severe atherosclerosis than control male mice as shown by the increased sizes of atherosclerotic lesion areas in the aorta, aortic arch, and thoracic aorta (Fig. 2*A*) and at the section of the aorta root (Fig. 2*B*). However, no significant difference was observed for the necrotic core areas between the two groups of mice (Fig. 2*C*), indicating that *ANRIL* plays a less important role in necrotic core development that is associated most often with apoptosis and secondary necrosis of foam cells and VSMCs (45). Interestingly, the signals of F4-80 immunostaining and Masson staining were significantly increased in atherosclerotic lesion areas in *ApoE*^*−/−*^*ANRIL* mice, indicating a prominent role of macrophages and collagen in the development of atherosclerosis in *ApoE*^*−/−*^*ANRIL* mice (Fig. 2, *D* and *E*). No significant difference was detected for body weight, serum triglyceride (TG), total cholesterol (TC), high-density lipoprotein cholesterol (HDL-c), and low-density lipoprotein cholesterol (LDL-c) between the two groups of mice (Fig. 2*F*). Similar results were obtained from female mice (Fig. S1, *A–F*). Together, these *in vivo* data establish *ANRIL* as a disease-causing gene for atherosclerosis.

RT-qPCR analysis showed that the expression of inflammation-related genes *Il1b*, *Tnf-a*, and *Il6* was significantly increased, while anti-inflammatory gene *Il10* was downregulated in the atherosclerotic lesions of *ApoE*^*−/−*^*ANRIL* mice (Fig. 2*G*). ELISA showed that serum IL-1β was also significantly increased in *ApoE*^*−/−*^*ANRIL* mice (Fig. 2*H*). The data suggest that *ANRIL* overexpression induced inflammation in *ApoE*^*−/−*^*ANRIL* mice.

### *ANRIL* overexpression impairs lysosomal pH homeostasis by upregulating TMEM106B in macrophages

**Figure 3:**
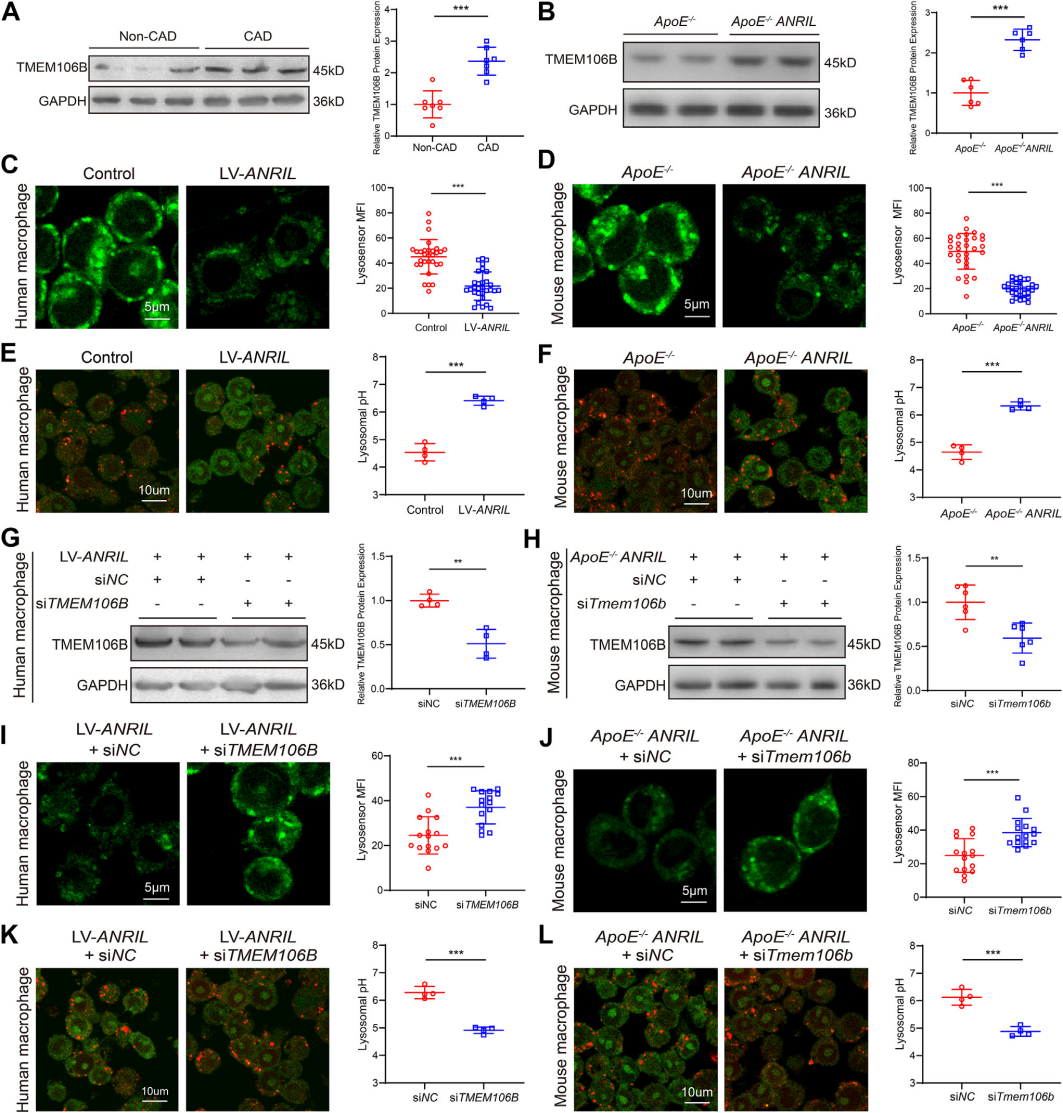
***ANRIL* increases lysosomal pH in macrophages by upregulating *TMEM106B* expression.***A*, Western blot analysis for TMEM106B in human CAD patients. n = 7. *B*, Western blot analysis for TMEM106B in atherosclerotic lesions of mice. n = 6. *C*, human PBMC-induced macrophages with *ANRIL* overexpression (LV-*ANRIL*) stained for Lysosensor. n = 30. *D*, mouse primary macrophages stained for Lysosensor. n = 30. *E*, lysosomal pH measurement in human PBMC-induced macrophages infected with LV-*ANRIL* or control viruses with a combination of pH-insensitive (TRITC) and sensitive (FITC) dyes. n = 4 samples with >50 cells counted in each sample. *F*, lysosomal pH measurement in macrophages isolated from *ApoE*^*−/−*^ and *ApoE*^*−/−*^*ANRIL* mice with a combination of pH-insensitive (TRITC) and sensitive (FITC) dyes. n = 4 samples with >50 cells counted in each sample. *G*, Western blot analysis for TMEM106B in human PBMC-induced macrophages with *ANRIL* overexpression transfected with si*TMEM106B* or si*NC*. n = 4. *H*, Western blot analysis for TMEM106B in mouse primary macrophages transfected with si*Tmem106b* or si*NC*. n = 6. *I*, human PBMC-induced macrophages transfected with si*TMEM106B* or si*NC* and stained for Lysosensor. n = 15. *J*, mouse primary macrophages transfected with si*Tmem106b* or si*NC* and stained for Lysosensor. n = 15. *K*, lysosomal pH measurement in human PBMC-induced macrophages with *ANRIL* overexpression transfected with si*NC* or si*TMEM106B* with a combination of pH-insensitive (TRITC) and sensitive (FITC) dyes. n = 4 samples with >50 cells counted in each sample. *L*, lysosomal pH measurement in macrophages isolated from *ApoE*^*−/−*^*ANRIL* mice and transfected with si*NC* or si*Tmem106b* with a combination of pH-insensitive (TRITC) and sensitive (FITC) dyes. n = 4 samples with >50 cells counted in each sample. ∗∗*p* < 0.01, ∗∗∗*p* < 0.001. Unpaired two-tailed *t* test.

We previously reported that *ANRIL* regulated endothelial cell functions associated with atherosclerosis by upregulating multiple genes, including *TMEM106B* (14, 31). Consistent with these results, RT-qPCR analysis showed that compared with control mice, *Tmem106b* was significantly upregulated in atherosclerotic aortic tissues of *ApoE*^*−/−*^*ANRIL* mice, while other genes showed no significant differences (Fig. S2). Western blotting further showed that TMEM106B expression was significantly increased in atherosclerotic aortic tissues from human CAD patients (Fig. 3*A*) and *ApoE*^*−/−*^*ANRIL* mice (Fig. 3*B*) compared with controls.

TMEM106B is a lysosomal membrane protein that was shown to regulate lysosomal pH homeostasis in neuronal cells (23, 40). Based on this finding, we hypothesized that *ANRIL* modulated lysosomal pH in the cardiovascular system by regulating TMEM106B expression. To test the hypothesis, we used Lysosensor, a lysosomal pH-sensitive dye, to examine the acid-base status of lysosomes in different cardiovascular cells infected with lenti-*ANRIL* viruses (LV-*ANRIL*) (Fig. S3*A*). Western blotting analysis showed that overexpression of *ANRIL* increased TMEM106B expression in human primary macrophages, primary human coronary artery endothelial cells, and primary human coronary artery smooth muscle cells (Fig. S3*B*). However, Lysosensor staining showed that lysosomal pH was significantly increased (indicating a decrease in mean fluorescence intensity) in human primary macrophages with *ANRIL* overexpression (Fig. 3*C*), but not in human coronary artery endothelial cells (Fig. S3*C*) or human coronary artery smooth muscle cells (Fig. S3*D*) with *ANRIL* overexpression. Lysosensor staining of primary macrophages isolated from mice also showed that lysosomal pH was significantly increased in *ApoE*^*−/−*^*ANRIL* mice compared to control mice (Fig. 3*D*), suggesting that *ANRIL* regulates lysosomal pH. To validate this conclusion, we used a combination of pH-insensitive (TRITC) and pH-sensitive (FITC) dye to detect the pH in lysosomes. The fluorescence intensity of TRITC was independent of lysosomal pH, while the fluorescence intensity of FITC was positively correlated with lysosomal pH. The fitting of FITC/TRITC ratios to the calibration group was used to calculate the lysosomal pH of the experimental group (Fig. S4). Our data showed that the baseline lysosomal pH was about 4.6 in macrophages, but *ANRIL* overexpression significantly increased the lysosomal pH to about 6.3 in human and mouse primary macrophages (Fig. 3, *E* and *F*). Interestingly, TMEM106B knockdown successfully reduced TMEM106B expression in human (Fig. 3*G*) and mouse primary macrophages (Fig. 3*H*) and reversed the increased lysosomal pH caused by *ANRIL* overexpression in both human (Fig. 3, *I* and *K*) and mouse primary macrophages (Fig. 3, *J* and *L*). Together, these data suggest that *ANRIL* overexpression increases lysosomal pH homeostasis by upregulating TMEM106B in macrophages.

### TMEM106B knockdown reverses inflammation and autophagic phenotypes caused by *ANRIL* overexpression

**Figure 4:**
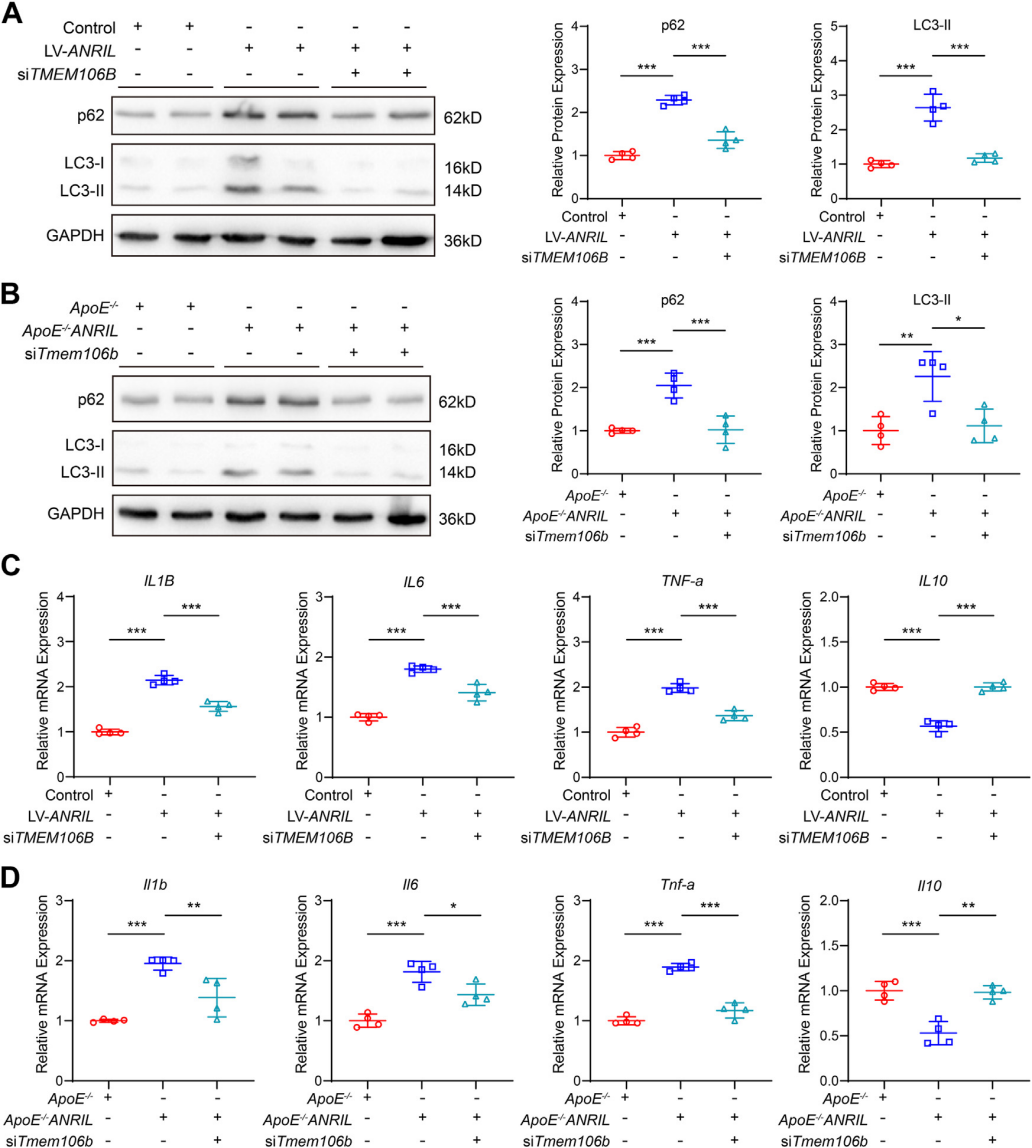
**Knockdown of *TMEM106B* reverses autophagic and inflammatory phenotypes caused by *ANRIL* overexpression.***A*, Western blot analysis for p62 and LC3-II in human primary macrophages. n = 4. *B*, Western blot analysis for p62 and LC3-II in mouse primary macrophages. n = 4. *C*, RT-qPCR analysis for *IL1B*, *IL6*, *TNF-a*, and *IL10* in human primary macrophages. n = 4. *D*, RT-qPCR analysis for *Il1b*, *Il6*, *Tnf-a*, and *Il10* in mouse primary macrophages. ∗*p* < 0.05, ∗∗*p* < 0.01, ∗∗∗*p* < 0.001. One-way ANOVA.

Because TMEM106B modulates lysosomal pH homeostasis and the lysosome plays an important role in the autophagy–lysosomal pathway (46), we determined whether *ANRIL* regulated autophagy through TMEM106B. WB analysis showed that the expression of p62 and LC3-II was significantly increased in human primary macrophages (Fig. 4*A*) and mouse primary macrophages (Fig. 4*B*) with *ANRIL* overexpression, which was reversed by *TMEM106B* knockdown (Fig. 4, *A* and *B*). The data suggest that *ANRIL* overexpression inhibits autophagy through TMEM106B. RT-qPCR analysis showed that the expression of inflammation markers *IL1B*, *IL6*, and *TNF-a* was upregulated, but *IL10* was downregulated by *ANRIL* overexpression in both human primary macrophages (Fig. 4*C*) and mouse primary macrophages (Fig. 4*D*). These effects were reversed by *TMEM106B* knockdown (Fig. 4, *C* and *D*). These results suggest that *TMEM106B* knockdown reverses the autophagic and inflammatory phenotypes caused by *ANRIL* overexpression.

### *ANRIL* increases TMEM106B expression by reducing *miR-181b-5p* expression

**Figure 5:**
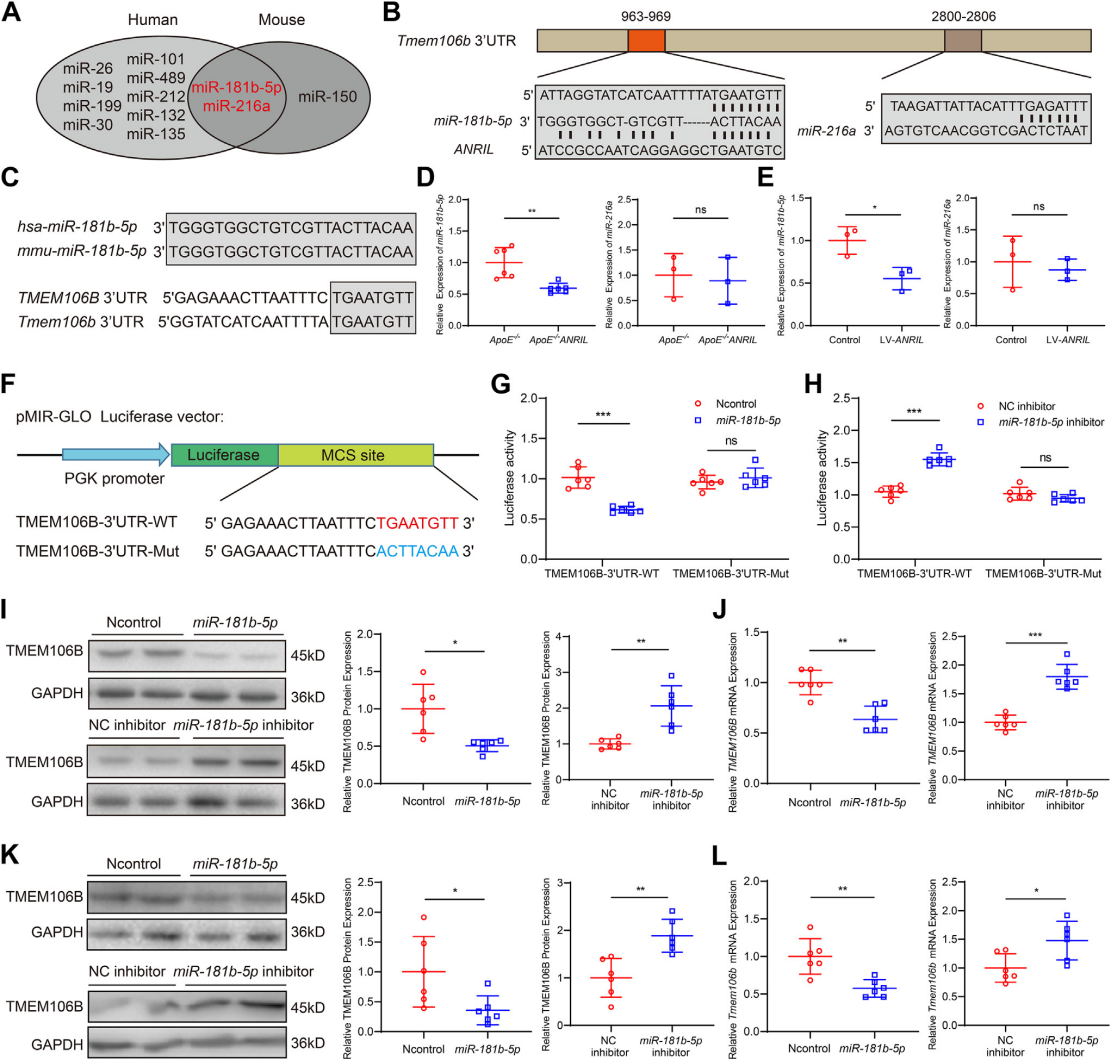
***ANRIL* upregulates *TMEM106B* expression *via* reducing *miR-181b-5p*.***A*, intersection of human and mouse miRNAs that bind to *Tmem106b* 3′UTR. *B*, *miR-181b-5p* has complementary pairing sequences with *ANRIL* and *Tmem106b* 3′UTR. *C*, homology analysis of *miR-181b-5p* and *TMEM106B*-3′UTR in humans and mice. *D*, RT-qPCR analysis showed the expression of *miR-181b-5p* and *miR-216a* in mice. n = 3 to 6. *E*, RT-qPCR analysis showed the expression of *miR-181b-5p* and *miR-216a* in human primary macrophages. n = 3. *F*, schematic diagram showing pMIR-TMEM106B-3′UTR-WT or pMIR-TMEM106B-3′UTR-Mut reporters with the *miR-181b-5p*–binding site. *G* and *H*, luciferase activity of TMEM106B-3′UTR-WT or TMEM106B-3′UTR-Mut reporters in the presence of *miR-181b-5p* mimics *versus* Ncontrol or *miR-181b-5p* inhibitor *versus* NC inhibitor. n = 6. *I* and *J*, Western blot analysis and RT-qPCR analysis for TMEM106B in THP1-induced macrophages (100 ng/ml PMA for 48h) transfected with *miR-181b-5p* mimics or *miR-181b-5p* inhibitor. n = 6. *K* and *L*, Western blot analysis and RT-qPCR analysis for TMEM106B in mouse RAW264.7 macrophages transfected with *miR-181b-5p* mimics or *miR-181b-5p* inhibitor. n = 6. ∗*p* < 0.05, ∗∗*p* < 0.01, ∗∗∗*p* < 0.001, ns, not significant. *D*, *E*, *I*, and *J*, (*right*), (*K* and *L*) unpaired two-tailed *t* test; (*G* and *H*) two-way ANOVA; (*J*) (*left*) nonparametric test.

To identify the molecular mechanism by which *ANRIL* regulates TMEM106B, we hypothesized that *ANRIL* increased the expression of *TMEM106B* by reducing the expression of microRNAs. Bioinformatic analysis (http://www.targetscan.org/) identified 11 microRNAs at the 3′UTR of human *TMEM106B* mRNA and three microRNAs at the 3′UTR of mouse *Tmem106b* mRNA (Fig. S5). *MiR-181b-5p* and *miR-216a* can bind to the *TMEM106B* 3′UTR region in both humans and mice (Fig. 5*A*), but only *miR-181b-5p* has sequences that can also pair with *ANRIL* (Fig. 5*B*) (47, 48, 49, 50). Homology analysis showed that the sequences of *miR-181b-5p* were completely conserved in humans and mice, and the binding region (seed sequences) of *Tmem106b* 3′UTR to *miR-181b-5p* was also conserved between humans and mice (Fig. 5*C*). RT-qPCR analysis showed that the expression of *miR-181b-5p*, but not *miR-216a*, was significantly decreased in *ApoE*^*−/−*^*ANRIL* mice (Fig. 5*D*) and human primary macrophages with *ANRIL* overexpression (Fig. 5*E*).

To further demonstrate the regulation of *TMEM106B* by *miR-181b-5p*, we constructed two luciferase reporters, including *pMIR-TMEM106B-WT-3′UTR* with *TMEM106B* 3′UTR containing the WT *miR-181b-5p* binding site and *pMIR-TMEM106B-Mut-3′UTR* with the *miR-181b-5p* binding site mutated (Fig. 5*F*). Luciferase assays showed that for *pMIR-TMEM106B-WT-3′UTR*, *miR-181b-5p* significantly decreased the luciferase activity compared with Ncontrol; however, this effect was not observed with mutant *pMIR-TMEM106B-Mut-3′UTR* (Fig. 5*G*). A *miR-181b-5p* inhibitor significantly increased the luciferase activity from *pMIR-TMEM106B-WT-3′UTR* but not from mutant *pMIR-TMEM106B-Mut-3′UTR* (Fig. 5*H*). Western blotting and RT-qPCR analysis showed that *TMEM106B* expression was significantly decreased by *miR-181b-5p* but increased by *miR-181b-5p* inhibitor in human THP1-induced macrophages (Fig. 5, *I* and *J*) and mouse RAW264.7 macrophages (Fig. 5, *K* and *L*). These results indicate that *ANRIL* reduces *miR-181b-5p* expression, which leads to increased TMEM106B expression.

### TMEM106B increases lysosomal pH by interacting with NHE1

**Figure 6:**
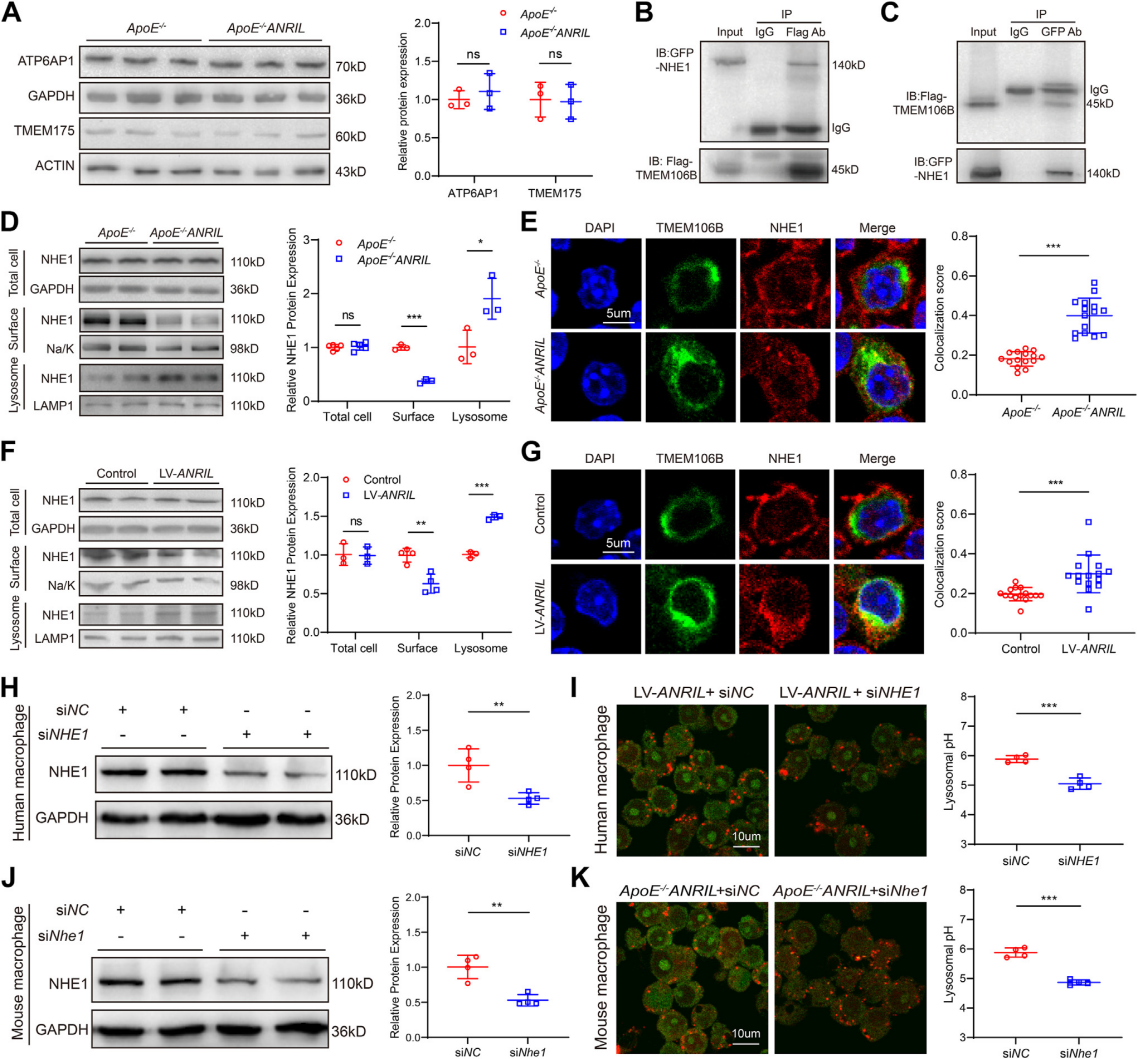
**TMEM106B interacts with NHE1 and colocalization of NHE1 and TMEM106B in lysosomes after *ANRIL* overexpression.***A*, Western blot analysis for ATP6AP1 and TMEM175 in mouse primary macrophages. n = 3. *B* and *C*, Co-IP for the interaction between TMEM106B and NHE1 in HEK293 cells. *D*, Western blot analysis for NHE1 in total lysates, cell surface lysates, and lysosomal lysates of mouse primary macrophages. n = 3 to 5. *E*, colocalization of NHE1 and TMEM106B in the lysosomes of mouse primary macrophages. n = 15. *F*, Western blot analysis for NHE1 in total lysates, cell surface lysates, and lysosomal lysates of human PBMC-induced primary macrophages. n = 3 to 4. *G*, colocalization of NHE1 and TMEM106B in the lysosomes of human PBMC-induced primary macrophages. n = 15. *H*, Western blot analysis for NHE1 in human PBMC-induced macrophages that were first infected with LV-*ANRIL* and then transfected with si*NC* or si*NHE1*. n = 4. *I*, lysosomal pH measurements in human PBMC-induced macrophages treated as in (*H*). n = 4 samples with >50 cells counted in each sample. *J*, Western blot analysis for NHE1 in macrophages isolated from *ApoE*^*−/−*^*ANRIL* mice and transfected with si*NC* or si*Nhe1*. n = 4. *K*, lysosomal pH measurements in *ApoE*^*−/−*^*ANRIL* macrophages treated as in (*J*). ∗*p* < 0.05, ∗∗*p* < 0.01, ∗∗∗*p* < 0.001, ns, not significant. *A*, *D*, and *F*, multiple *t* test; (*E* and *G–K*) unpaired two-tailed *t* test.

Knockdown of *TMEM106B* was shown to interact with ATP6AP1 (lysosomal V-ATPase AP1) and decrease the level of ATP6AP1, resulting in increased lysosomal pH in cortical neurons due to its function as an influx proton channel (40). In addition to the influx proton channel V-ATPase, there is a proton-selective efflux channel TMEM175 on the lysosome, which is also important for maintaining lysosomal pH homeostasis (38). However, the expression of ATP6AP1 and TMEM175 did not show any significant difference between *ApoE*^*−/−*^*ANRIL* mice and control mice (Fig. 6*A*). Furthermore, as ATP6AP1 interacts with TMEM106B, we carried out experiments to investigate whether *ANRIL* increases lysosomal pH through ATP6AP1. We knocked *ATP6AP1* down or overexpressed *ATP6AP1* in human macrophages (Fig. S6, *A* and *C*) and mouse macrophages (Fig. S6, *E* and *G*) and then measured lysosomal pH. Lysosomal pH analysis showed that neither knockdown nor overexpression of *ATP6AP1* reversed the increase of lysosomal pH caused by *ANRIL* overexpression in both human macrophages (Fig. S6, *B* and *D*) and mouse macrophages (Fig. S6, *F* and *H*). These results suggest that *ATP6AP1* is unlikely to be responsible for the increased lysosomal pH detected in macrophages with overexpression of *ANRIL*.

To determine how the *ANRIL–TMEM106B* molecular pathway increases lysosomal pH in macrophages, we searched for other candidate proteins that interact with TMEM106B by querying the BioGRID database (https://downloads.thebiogrid.org/BioGRID/Release-Archive/BIOGRID-4.3.196/). NHE1 became an outstanding candidate due to its role in regulating intracellular pH as a sodium/hydrogen exchanger present on the cell surface. The co-immunoprecipitation (Co-IP) assay showed that TMEM106B indeed interacted with NHE1. Flag-TMEM106B successfully precipitated GFP-NHE1, whereas negative control IgG did not (Fig. 6*B*) and *vice versa* (Fig. 6*C*). To confirm this result, we repeated Co-IP experiments with the samples without the bait expressed. Western blotting showed that the anti-GFP antibody pulled Flag-NHE1 down from cells with co-expression of GFP-TMEM106B and Flag-NHE1, but not from cells cotransfected with the GFP vector and Flag-NHE1 plasmid (Fig. S7*A*). Similarly, Western blotting analysis showed that the anti-Flag antibody pulled GFP-TMEM106B down from cells transfected with Flag-NHE1 and GFP-TMEM106B plasmids, but not from cells transfected with the Flag vector and GFP-TMEM106B plasmid (Fig. S7*B*).

TMEM106B is an integral type 2 glycosylated transmembrane protein with the C-terminal domain located within the lysosomal lumen and the N-terminal domain within the cytosol, but NHE1 is assumed to be located on the cell surface. It was, therefore, interesting to investigate how the interaction between TMEM106B and NHE1 modulates the cellular localization of NHE1. Western blotting showed that the amount of NHE1 in total cellular lysates was similar in primary macrophages isolated from *ApoE*^*−/−*^*ANRIL* mice and control mice (Fig. 6*D*) and in human macrophages with and without overexpression of *ANRIL* (Fig. 6*F*). It was surprising to find that NHE1 was also detected in lysosomal lysates and significantly upregulated in lysosomal lysates in *ApoE*^*−/−*^*ANRIL* macrophages (Fig. 6*D*) and in human macrophages with *ANRIL* overexpression (Fig. 6*F*). Moreover, NHE1 expression was downregulated on the cell surface of macrophages with overexpression of *ANRIL* (Fig. 6, *D* and *F*). These results were further validated using co-immunostaining for NHE1 and TMEM106B in primary macrophages with overexpression of *ANRIL*. In macrophages without overexpression of *ANRIL*, NHE1 was localized on the cell surface, but was colocalized with TMEM106B to lysosomes in macrophages with *ANRIL* overexpression (Fig. 6, *E* and *G*). NHE1 knockdown successfully reduced NHE1 expression in both human (Fig. 6*H*) and mouse macrophages (Fig. 6*J*). Lysosomal pH analysis showed that *NHE1* knockdown significantly reduced lysosomal pH in human and mouse macrophages with *ANRIL* overexpression (Fig. 6, *I* and *K*). These data suggest that knockdown of *NHE1* can reverse the increased lysosomal pH in macrophages with *ANRIL* overexpression. Together, these data suggest that the interaction between TMEM106B and NHE1 promotes the sequestering of NHE1 from plasma membranes to lysosomal membranes, resulting in increased lysosomal pH in macrophages.

### Truncation and point mutation analyses define the TMEM106B–NHE1 interaction domain as F115 and F117 on TMEM106B

**Figure 7:**
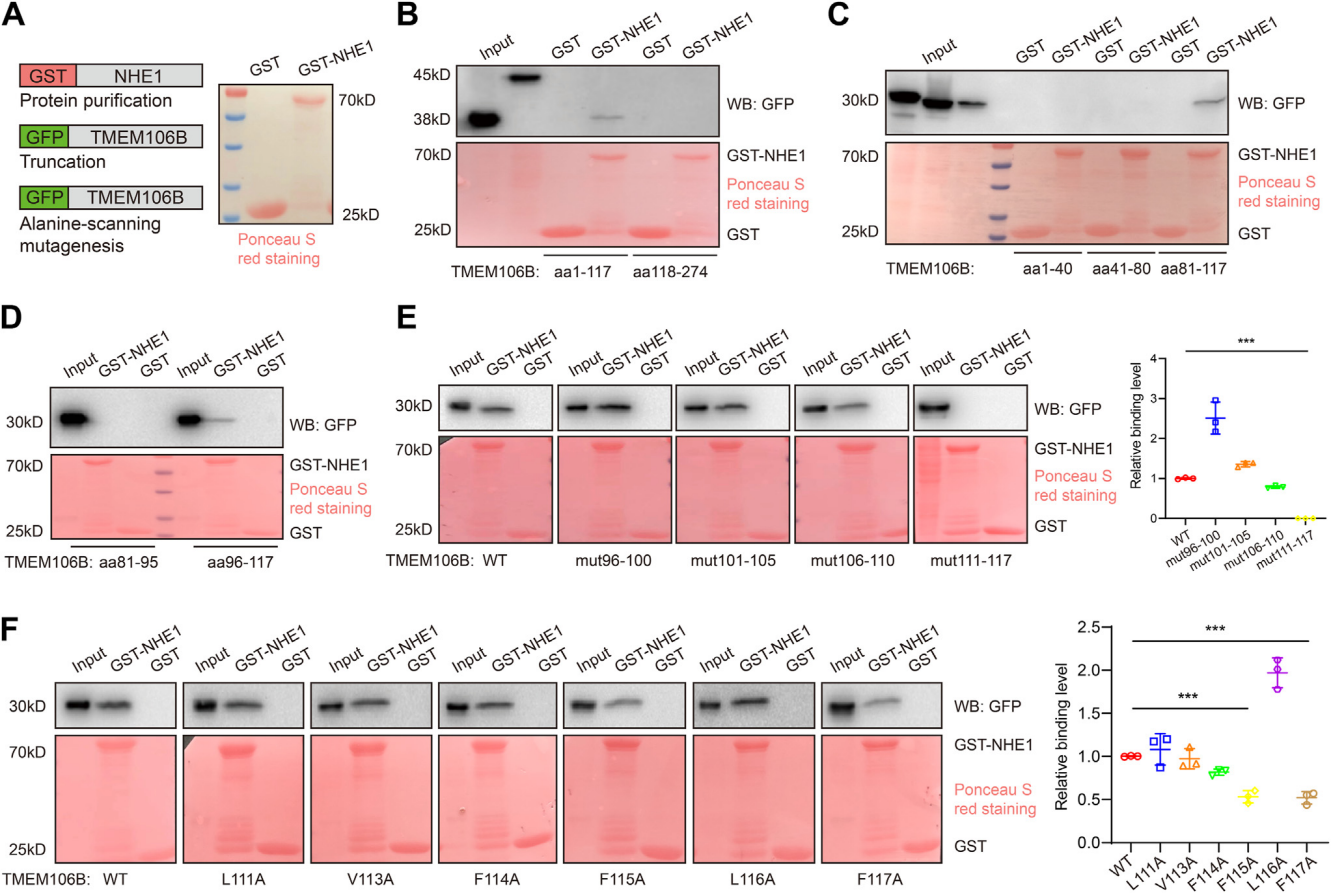
**Truncation and point mutation analyses define the TMEM106B–NHE1 interaction domain on TMEM106B.***A*, purification of GST-NHE1 protein. *B*, GST pull-down assays identified the TMEM106B–NHE1 interaction region as aa1-117 of TMEM106B. *C*, GST pull-down assays identified the TMEM106B–NHE1 interaction region as aa81-117 of TMEM106B. *D*, GST pull-down assays identified the TMEM106B–NHE1 interaction region as aa96-117 of TMEM106B. *E*, amino acids 111-117 of TMEM106B were the critical residues for the TMEM106B–NHE1 interaction. n = 3. *F*, F115 and F117 of TMEM106B were the critical residues for the TMEM106B–NHE1 interaction. n = 3. ∗∗∗*p* < 0.001. One-way ANOVA.

To define the molecular basis for the interaction between TMEM106B and NHE1, we characterized the interaction between the glutathione-S-transferase (GST)-NHE1 fusion protein and different mutant GFP-TMEM106B proteins (Fig. 7*A*). GST pull-down showed that the aa1-117 region (amino acids 1-117) of TMEM106B, but not the aa118-274 region, interacted with NHE1 (Fig. 7*B*). Further mutation analysis showed that aa81-117 of TMEM106B interacted with NHE1 (Fig. 7*C*). Additional analysis showed that aa96-117 of TMEM106B interacted with NHE1 (Fig. 7*D*). Then, alanine-scanning mutagenesis of the aa96-117 domain was performed to show that NHE1 interacted with TMEM106B-LYVMA96-100AAAAA, TMEM106B-SVFVC101-105AAAAA, or TMEM106B-LLLSG106-110AAAAA, but not with TMEM106B-LAVFFLF111-117AAAAAAA (Fig. 7*E*). The data indicate that aa111-117 of TMEM106B is the binding domain for NHE1. Furthermore, the interaction of NHE1 with TMEM106B F115A and F117A mutants was significantly weaker than that with WT, L111A, V113A, F114A, or L116A mutants (Fig. 7*F*). The data indicate that amino acids F115 and F117 of TMEM106B are critical for NHE1 binding.

### Truncation and point mutation analyses define the TMEM106B–NHE1 interaction domain as I537, C538, and G539 on NHE1

**Figure 8:**
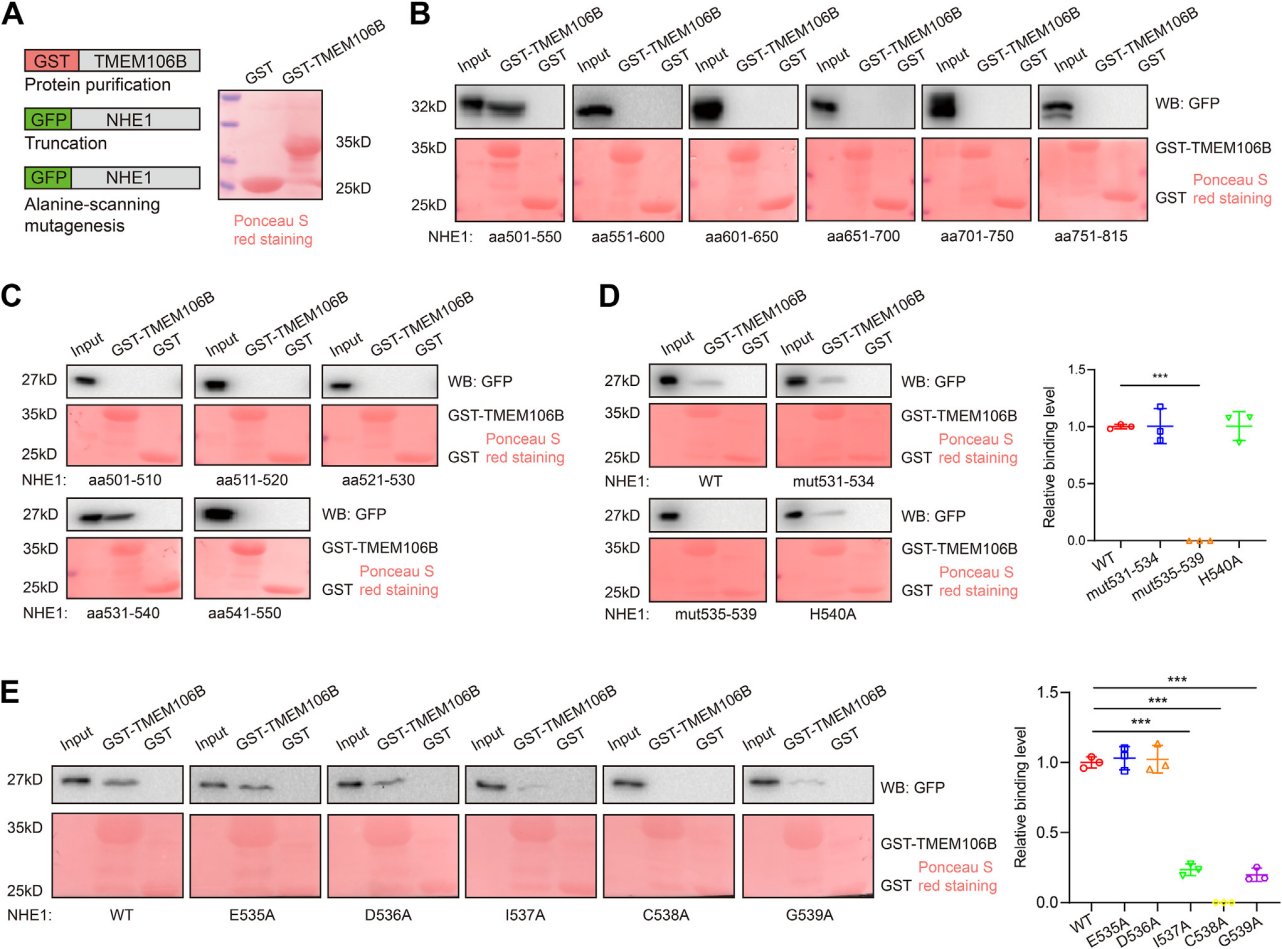
**Truncation and point mutation analyses define the TMEM106B–NHE1 interaction domain on NHE1.***A*, purification of GST-TMEM106B protein. *B*, GST pull-down assays identified the TMEM106B–NHE1 interaction region as aa501-550 of NHE1. *C*, GST pull-down assays identified the TMEM106B–NHE1 interaction region as aa531-540 of NHE1. *D*, amino acids 535-539 were the critical residues for the TMEM106B–NHE1 interaction. n = 3. *E*, I537, C538, and G539 of NHE1 were the critical residues for the TMEM106B–NHE1 interaction. n = 3. ∗∗∗*p* < 0.001. One-way ANOVA.

To identify the TMEM106B-binding site on NHE1, we characterized the interaction between the GST–TMEM106B fusion protein and different mutants of the GFP-NHE1 fusion protein (Fig. 8*A*). GST pull-down showed that the aa501-550 region of NHE1 interacted with TMEM106B (Fig. 8*B*). Additional analysis showed that aa531-540 of NHE1 interacted with TMEM106B (Fig. 8*C*). Alanine-scanning mutagenesis showed that TMEM106B interacted with NHE1-LTGI531-534AAAA and NHE1-H540A, but not with NHE1-EDICG535-539AAAAA (Fig. 8*D*). The data indicate that aa535-539 of NHE1 is the TMEM106B-binding domain. Moreover, TMEM106B interacted weakly with the NHE1 I537A and G539A mutants, but barely with the C538A mutant (Fig. 8*E*). The data indicate that amino acids I537, C538, and G539 of NHE1 are the critical amino acid residues responsible for the interaction between NHE1 and TMEM106B.

### A TMEM106B peptide antagonist normalizes the lysosomal pH of macrophages with overexpression of *ANRIL* by blocking the interaction between TMEM106B and NHE1

**Figure 9:**
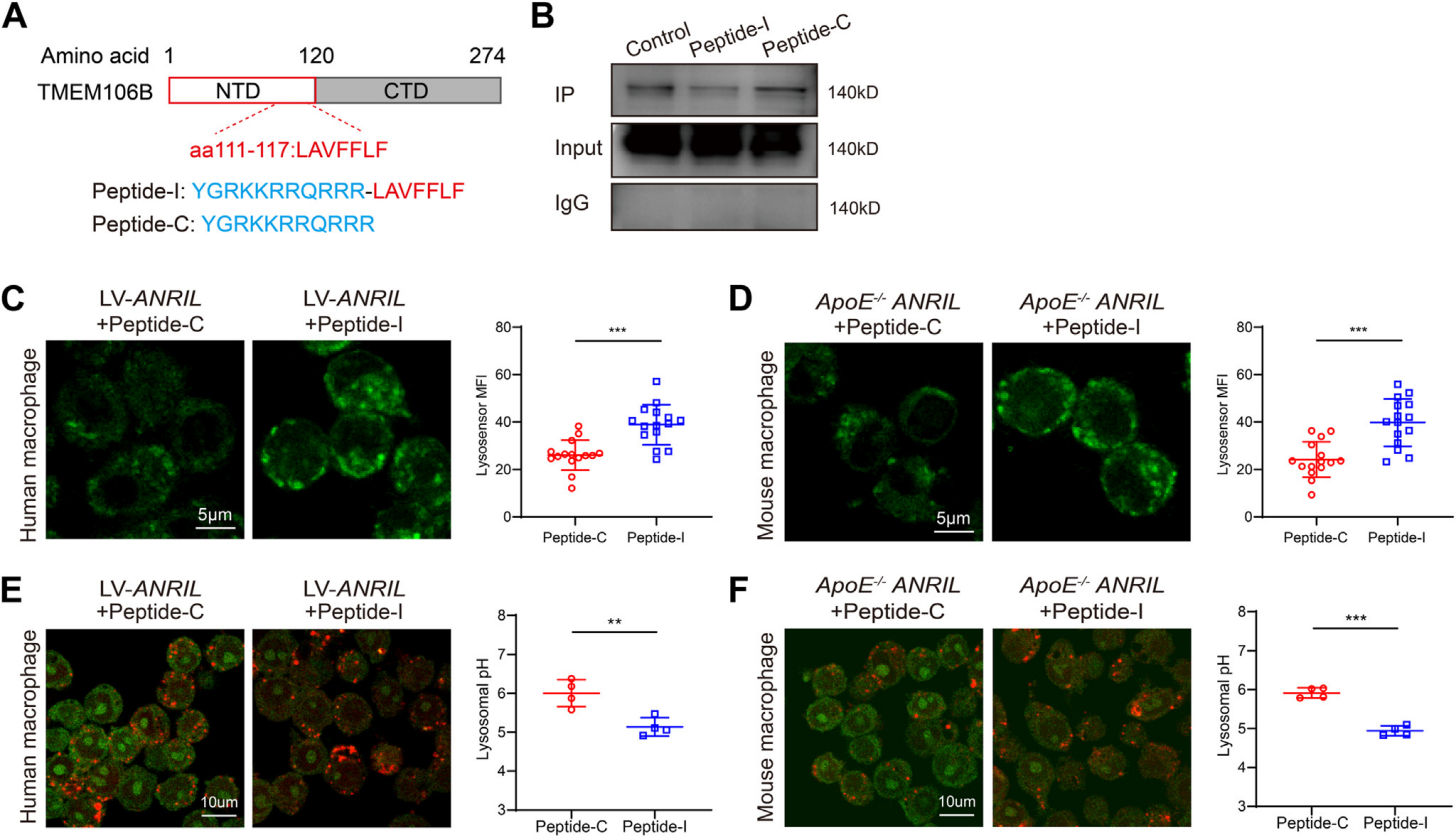
**TMEM106B peptide-I restores lysosomal pH homeostasis by inhibiting TMEM106B–NHE1 interaction.***A*, sequences of peptides. *B*, co-IP showing the inhibitory effect of peptide-I on the TMEM106B–NHE1 interaction. *C*, lysosensor staining showed that peptide-I normalized lysosomal pH homeostasis in human primary macrophages. n = 15. *D*, lysosensor staining showed that peptide-I normalized lysosomal pH homeostasis in mouse primary macrophages. n = 15. *E*, lysosomal pH measurement in human PBMC-induced macrophages with *ANRIL* overexpression and treated with peptide-C or peptide-I with a combination of pH-insensitive (TRITC) and sensitive (FITC) dyes. n = 4 samples with >50 cells counted in each sample. *F*, lysosomal pH measurement in macrophages isolated from *ApoE*^*−/−*^*ANRIL* mice and treated with peptide-C or peptide-I with a combination of pH-insensitive (TRITC) and sensitive (FITC) dyes. n = 4 samples with >50 cells counted in each sample. ∗∗∗*p* < 0.001. Unpaired two-tailed *t* test.

To determine the pathophysiological importance of the interaction between TMEM106B and NHE1, we designed and synthesized an inhibitory TMEM106B peptide LAVFFLF fused with the membrane-penetrating sequence YGRKKRRQRRR (peptide-I) based on the interaction domain sequences of TMEM106B as defined above (Fig. 9*A*). Peptide-C YGRKKRRQRRR with only the membrane-penetrating sequence served as the negative control (Fig. 9*A*). Co-IP analysis showed that compared with peptide-C, peptide-I weakened the interaction between TMEM106B and NHE1 (Fig. 9*B*), which may be due to the competitive binding of NHE1 by a high concentration of peptide-I and leaves few NHE1 molecules to interact with TMEM106B. In both human primary macrophages with overexpression of *ANRIL* and primary macrophages isolated from *ApoE*^*−/−*^*ANRIL* mice, treatments with peptide-I showed significantly reduced lysosomal pH compared to treatments with peptide-C (Fig. 9, *C* and *D*). The pH measurements with the combination of pH-insensitive (TRITC) and pH-sensitive (FITC) dye showed that peptide-I treatment significantly reduced the lysosomal pH from 6.0 to 5.0 in both human and mouse macrophages with *ANRIL* overexpression (Fig. 9, *E* and *F*). The data indicate that peptide-I normalized lysosomal pH in macrophages with the overexpression of *ANRIL* and suggest that the interaction between TMEM106B and NHE1 is involved in the regulation of lysosomal pH homeostasis (acidification) in macrophages.

### TMEM106B-F115A and TMEM106B-F117A mutations disrupt colocalization of NHE1 with TMEM106B on lysosomal membranes and reduces lysosomal pH

**Figure 10:**
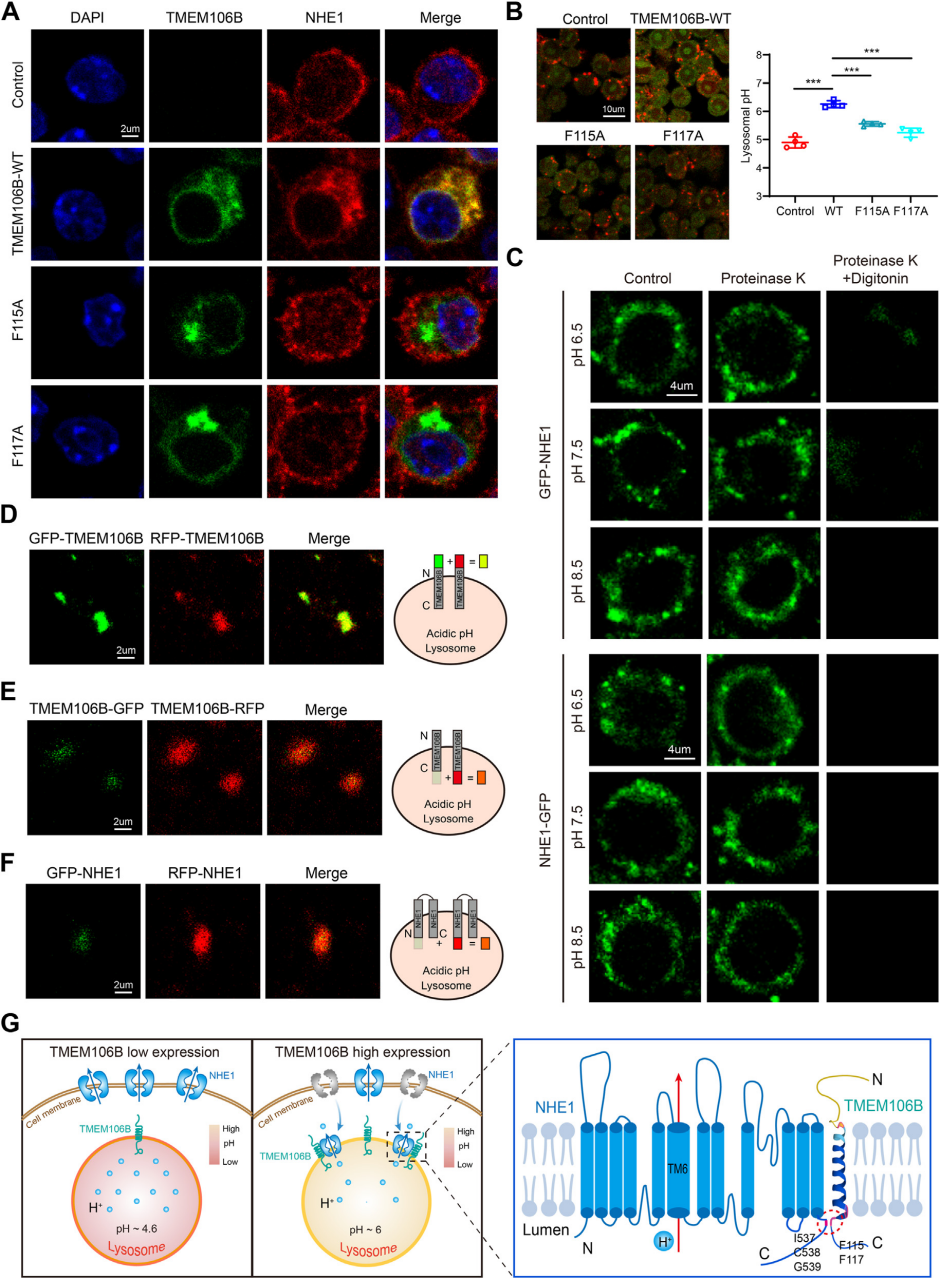
**Mutations F115A and F117A of TMEM106B impair cytoplasmic colocalization of TMEM106B and NHE1 and analysis of NHE1 topology on lysosomal membranes.***A*, immunostaining showing colocalization of TMEM106B and NHE1, which was disrupted by TMEM106B-F115A and TMEM106B-F117A mutations. *B*, TMEM106B-F115A and TMEM106B-F117A mutations significantly inhibited the effect of TMEM106B-WT on lysosomal pH. n = 4 samples with >50 cells counted in each sample. *C*, macrophages were transfected with GFP-NHE1 or NHE1-GFP with GFP tagged at the N terminus or C terminus, respectively. Cells were treated with control PBS, proteinase K, and proteinase K plus digitonin, respectively, and fluorescence signals were detected under a confocal microscope. *D*, the lysosomes were isolated from the macrophages and imaged for analysis of the topology of N-terminal tagged GFP-TMEM106B and RFP-TMEM106B on lysosomal membranes. *E*, the lysosomes were isolated from the macrophages and imaged for analysis of the topology of C-terminal tagged TMEM106B-GFP and TMEM106B-RFP on lysosomal membranes. *F*, the lysosomes were isolated from the macrophages and imaged for analysis of the topology of N-terminal tagged GFP-NHE1 and RFP-NHE1 on lysosomal membranes. *G*, schematic diagram of NHE1 localization on plasma membranes and lysosomal membranes and effects on cellular and lysosomal pH. ∗∗∗*p* < 0.001. One-way ANOVA.

Our Co-IP and GST pull-down assays showed the interaction between NHE1 and TMEM106B and identified amino acids F115 and F117 as the interacting sites on TMEM106B (Figure 6, Figure 7, Figure 8). To further confirm the critical role F115 and F117 in the interaction between NHE1 and TMEM106B, we performed immunostaining analysis using macrophages transfected with TMEM106B-WT or mutant TMEM106B with mutation F115A or F117A. In macrophages without overexpression of TMEM106B, NHE1 is localized on plasma membranes (Fig. 10*A*). However, when TMEM106B was overexpressed, WT TMEM106B-WT was colocalized with NHE1 on lysosomal membranes (Fig. 10*A*), confirming that overexpression of TMEM106B promotes mislocalization of NHE1 from plasma membranes to lysosomal membranes. The colocalization between NHE1 and TMEM106B was disrupted by mutant TMEM106B-F115A or TMEM106B-F117A in macrophages, which results in relocalization of NHE1 onto cell membranes (Fig. 10*A*). Lysosomal pH analysis showed that overexpression of WT TMEM106B significantly increased lysosomal pH to 6.3 compared with the control group; however, the effect was reversed by mutant TMEM106B-F115A or TMEM106B-F117A (lysosomal pH was reduced to 5.5 and 5.2, respectively) (Fig. 10*B*). These results substantially strength our conclusion that NHE1 interacts with TMEM106B and that the NHE1–TMEM106B interaction plays an important role in regulating lysosomal pH.

We used Chai Discovery analysis (51) to analyze the 3D structures of WT and mutant TMEM106B and found that the transmembrane domain of TMEM106B forms an α-helix, which spans R92 to F116 (Fig. S8). The F115 and F117 residues are located at the C terminus of the transmembrane α-helix, and F117 appears to be located outside of the transmembrane α-helix. The TMEM106B transmembrane α-helix is 24 amino acids long, which is unusually long compared to the shortest transmembrane domain with 18 amino acids (52). This may make the transmembrane domain more flexible as a variable number of amino acid residues in the R92-F116 segment can serve as the transmembrane domain. The topology of TMEM106B transmembrane α-helix and unusually longer length may make it physically possible for F115 and F117 to interact with amino acid residues I537, C538, or G539 of NHE1. More interestingly, Chai Discovery predicted that the TMEM106B-F115A or TMEM106B-F117A mutants changed the conformation of the N terminus domain of TMEM106B (Fig. S8), suggesting that the interaction with NHE1 may change the conformation of TMEM106B.

### Topology of NHE1 on plasma membranes and lysosomal membranes

NHE1 is in a resting state when it is in a nonacidic pH environment (53, 54). When NHE1 senses an acidic pH environment, it changes to an outward-open state, and protons are transported outside of cells to maintain pH homeostasis in cells (53, 54). To demonstrate the topology of NHE1 on the plasma membrane of macrophages, we used a reported technique of fluorescence protease protection to show membrane protein topology in living cells (55). Compared with PBS treatment, proteinase K treatment did not affect the degradation of the N terminus of GFP-NHE1. However, cotreatment of cells with both proteinase K and digitonin resulted in markedly decreased fluorescence intensity of GFP-NHE1 (Fig. 10*C*). These data suggest that the topology of NHE1 is that the N terminus of NHE1 is located in the cytoplasm. Similar experiments were performed with NHE-GFP with GFP tagged at the C terminus, and the results showed that the C terminus of NHE1 is also located in the cytoplasm (Fig. 10*C*).

To understand the topology of NHE1 when mislocalized onto the lysosomal membrane in cells with overexpression of TMEM106B, we designed an experiment based on the properties of pH-sensitive GFP and pH-insensitive RFP to study the topology of NHE1 on the lysosomal membrane. The fluorescence intensity of RFP was independent of pH, while the fluorescence intensity of GFP was positively correlated with pH (56). We constructed and overexpressed GFP- and RFP-tagged NHE1 or TMEM106B at either the N terminus or C terminus in macrophages. The topology of TMEM106B is that the N terminus is located in the cytoplasm, and the C terminus is inside the lysosome lumen. As such, N-terminal–tagged GFP-TMEM106B and RFP-TMEM106B showed strong fluorescence signals because the GRP- and RFP-tags are outside of the lysosome (nonacidic pH) (Fig. 10*D*). On the other hand, C-terminal–tagged TMEM106B-GFP and TMEM106B-RFP showed different fluorescence signals. TMEM106B-GFP showed weaker signals because the GFP tag is inside the lysosome with acidic pH (Fig. 10*E*). Since the N terminus and C terminus of NHE1 are known to be located on the same side, we constructed fusion plasmids with the N-terminal GRP- or RFP-tags. GFP-NHE1 and RFP-NHE1 showed different fluorescence signals, and GFP-NHE1 showed weaker signals, indicating that the GFP tag is inside the lysosome with acidic pH (Fig. 10*F*). These results indicate that the topology of NHE1 on the lysosomal membrane is that both N terminus and C terminus are located inside the lysosome lumen (Fig. 10*G*). With this topology, when NHE1 is mislocalized onto the lysosomal membranes, NHE1 is activated by acidic pH (∼4.6) and turned into an outward-open state, which can transport protons outside of the lysosome, raising the pH in the lysosome.

## Discussion

This study is the first mouse study, to the best of our knowledge, to demonstrate the causal role of *ANRIL* in atherosclerosis *in vivo*. The 9p21.3 genetic locus for CAD was initially identified by GWAS in 2007 and later confirmed in numerous independent studies (4, 5, 6). Close to 100 CAD-associated variants at the 9p21.3 cluster into a 60-kb LD block (6). The locus contains multiple genes, including *MTAP*, *CDKN2A*, *CDKN2B*, and *ANRIL* (*CDKN2b-AS1*), all of which became the candidate disease-causing genes for CAD. Because the CAD-associated LD block overlaps with *ANRIL* only (6, 12), *ANRIL* became the strong candidate gene for CAD. Some *in vitro* studies and expression analysis from our group and other groups provided some evidence to support that *ANRIL* is the disease-causing gene for CAD at the locus (14, 15, 16). In particular, our previous study and multiple other independent studies showed that the expression of the full-length transcript of *ANRIL* (NR_003529) was significantly increased in human CAD arterial samples (13). This finding was further confirmed in this study (Fig. 1). To unequivocally show that *ANRIL* is the disease-causing gene for CAD, we created a transgenic line of mice with the expression level of *ANRIL* (NR_003529) comparable to that in human tissues and cells (Fig. 1). Atherosclerotic assays showed that *ANRIL* aggravated atherosclerosis in *ApoE*^*−/−*^ mice (Figs. 2 and S1). The data provide direct *in vivo* evidence to demonstrate that *ANRIL* plays a key role in atherosclerosis at the 9p21.3 CAD locus. As the 9p21.3 locus is the most important genetic locus for CAD and responsible for 13% of CAD cases, our finding at this locus represents an important advance for the CAD field as it provides a novel target for future genetic testing of CAD and the development of innovative strategies to prevent or treat CAD for a significant portion of CAD patients.

Further characterization of *ApoE*^*−/−*^*ANRIL* mice revealed the molecular mechanism by which *ANRIL* exerted its functional and regulatory roles. Our RT-qPCR analysis and/or Western blotting showed that *ANRIL* reduced the expression of *miR-181b-5p*, whereas reduced *miR-181b-5p* expression increased the expression of *TMEM106B* in *ApoE*^*−/−*^*ANRIL* mice (Figs. 3 and 5). The results were replicated in human primary macrophages with overexpression of *ANRIL* (Figs. 3 and 5). Regulation of *TMEM106B* expression by *miR-181b-5p* was also demonstrated using a luciferase reporter in combination with a *miR-181b-5p* mimic and inhibitor in human THP1-induced macrophages and mouse RAW264.7 macrophages (Fig. 5). Consistent with our finding of the causal role of *ANRIL* in atherosclerosis, genetic variants of TMEM106B significantly increased the risk of CAD and interacted with *ANRIL* variants to confer an epistatic effect on risk of CAD (31). For *miR-181b-5p*, one study showed that its serum level was reduced in acute stroke patients with atherosclerotic plaque, and injection of *miR-181b* agomir reduced atherosclerotic area and plaque vulnerability by modulating macrophage polarization to M2 macrophages in *ApoE*^*−/−*^ mice (57). These published results support our finding that *ANRIL*, *miR-181b-5p*, and *TMEM106B* form a regulatory axis that is involved in the development of atherosclerosis in *ApoE*^*−/−*^*ANRIL* mice, although the definitive role of *miR-181b-5p* and TMEM106B in atherosclerosis requires characterization using transgenic mice with knockout or overexpression of the two genes.

One interesting finding from the present study is that TMEM106B interacts with NHE1 to modulate lysosomal pH homeostasis in macrophages (Fig. 3). First, in *ApoE*^*−/−*^*ANRIL* mice, *ANRIL* overexpression increased lysosomal pH in macrophages by upregulating TMEM106B (Fig. 3). Second, biochemical characterization showed that TMEM106B interacted with NHE1, TMEM106B amino acids F115 and F117 were critical for NHE1 binding, and NHE1 amino acids I537, C538, and G539 were critical for its interaction with TMEM106B (Figs. 7 and 8). Immunostaining analysis showed that TMEM106B and NHE1 colocalized on lysosomes in macrophages with *ANRIL* overexpression (Fig. 6), further indicating the interaction between TMEM106B and NHE1. Third, the interaction between TMEM106B and NHE1 is required for the increased lysosomal pH change in both human and mouse macrophages. A TMEM106B peptide antagonist normalized the lysosomal pH of macrophages by blocking the interaction between TMEM106B and NHE1 (Fig. 9). Moreover, F115A and F117A, two mutations affecting the critical amino acids required for TMEM106B–NHE1 interaction, disrupted the colocalization between TMEM106B and NHE1 on lysosomal membranes (Fig. 10*A*) and reversed the increased lysosomal pH induced by the overexpression of TMEM106B-WT (Fig. 10*B*).

Our topological studies of NHE1 and TMEM106B suggest that the interaction of TMEM106B with mislocalized NHE1 on lysosomal membranes is physically possible. Analyses of pH-sensitive GFP-tagged TMEM106B and pH-insensitive RFP-tagged TMEM106B showed that the topology of TMEM106B is that its N terminus is located in cytoplasm and the C terminus is localized inside the lysosome lumen (Fig. 10, *D* and *E*). Chai Discovery analysis suggests that F115 and F117, two critical TMEM106B amino acids required for TMEM106B–NHE1 interaction, are located inside the lysosomal lumen (Figs. 10*G* and S8). Topological studies showed that the topology of NHE1 on the lysosomal membrane is that both the N terminus and C terminus are located inside the lysosome lumen. With such a topology, NHE1 amino acid residues I537, C538, or G539 are also located inside the lysosomal lumen when NHE1 is mislocalized onto lysosomal membranes (Figs. 10*G* and S8). These data suggest that the interaction domains on NHE1 and TMEM106B are both located inside the lysosomal lumen, which makes the interaction between the two proteins physically possible.

With regard to the issue about the mechanism of the topology switch of NHE1 on the plasma membrane and lysosomal membrane, it may be due to the spatial localization of TMEM106B on lysosomal membranes, which has the N terminus located outside of the lysosome and the C terminus containing the TMEM106B–NHE1 interaction domain located inside the lysosome. Because the NHE1-interaction domain on TMEM106B is located inside the lysosome (Fig. 10*G*), the interaction domain on NHE1 needs to be located inside the lysosome so that the two proteins are located near each other. This requires that the C terminus of NHE1 with the TMEM106B interaction domain is located in the lysosomal lumen. Otherwise, the interaction domains on NHE1 and TMEM106B are separated by lysosomal membranes, which makes the interaction physically impossible. When TMEM106B forms a protein complex with NHE1 and is inserted into the lysosomal membrane, it pulls NHE1 into the lysosomal membrane. It is well-known that cell surface receptors, including EGFR, PD-1, and ASGPR, are trafficked from plasma membranes to the lysosome for degradation. However, to the best of our knowledge, NHE1 may be the first membrane protein that can be relocalized to the lysosomal membrane with a topology switch to execute important new functions.

Inconsistent results were reported on the regulation of lysosomal pH by TMEM106B knockdown and overexpression. Some studies showed that overexpression of *TMEM106B* led to increased lysosomal pH in HeLa cells and primary hippocampal neurons (23, 41). Similarly, our results in this study showed that in both human and mouse macrophages, *ANRIL* overexpression increased *TMEM106B* expression, which led to the increase of lysosomal pH (Fig. 3). On the other hand, loss of *TMEM106B* also resulted in an increase of lysosomal pH in HeLa cells, primary cultured cortical neurons, and BV2 cells (40, 58, 59). In contrast, Feng *et al.* showed that overexpression of *TMEM106B* reduced lysosomal pH in HEK293T cells (39). The conflicting results may be due to differences of cells used in different studies. A previous study showed that *TMEM106B* overexpression increased lysosomal pH in primary cultured cortical neurons due to its interaction with V-ATPase AP1 encoded by *ATP6AP1* and downregulation of the expression of the lysosomal V-ATPase, that is, V-ATPase AP1 or ATP6AP1 (40). However, we showed that ATP6AP1 was not responsible for the increased lysosomal pH in macrophages with the overexpression of *ANRIL* and *TMEM106B*. First, we found that *ANRIL* overexpression did not affect the expression of *ATP6AP1* (Fig. 6*A*). Second, neither knockdown nor overexpression of *ATP6AP1* reversed the increased lysosomal pH caused by *ANRIL* overexpression in both human and mouse macrophages (Fig. S6). Instead, our Co-IP and GST pull-down analyses demonstrated that NHE1 interacted with TMEM106B (Figure 6, Figure 7, Figure 8), and our immunostaining showed that TMEM106B was colocalized with NHE1 in the cytoplasm, and F115A and F117A mutation that disrupted the TMEM106B–NHE1 interaction impaired colocalization of NHE1 and TMEM106B on lysosomes and decreased lysosomal pH in macrophages (Fig. 10). Knockdown of NHE1 reversed the lysosome acidification in macrophages with the overexpression of *ANRIL* (Fig. 5). These results suggest that overexpression of *TMEM106B* caused by *ANRIL* increases lysosomal pH *via* NHE1 rather than ATP6AP1.

Our study defines NHE1 as a new proton (H^+^) efflux channel for lysosomes (Fig. 6). Topological studies showed that both the N terminus and C terminus of NHE1 are located inside the lysosome lumen when mislocalized onto lysosomal membranes. In this state, NHE1 may sense an acidic pH environment in lysosomes, transitions from a resting state to an outward-open state, and transports protons outside of lysosomes to increase lysosomal pH. Lysosomal pH homeostasis is maintained by the influx and efflux of protons. The V-ATPase is a well-established and the sole reported proton influx channel on lysosomes. For channel-mediated proton efflux, a recent 2022 study established TMEM175 as a new proton-selective efflux channel on lysosomes (38). In this study, we found that NHE1 could interact with TMEM106B, and the TMEM106B–NHE1 interaction increased lysosomal pH by promoting colocalization of NHE1 and TMEM106B onto lysosomes in macrophages (Fig. 6). NHE1 is the well-known Na^+^/H^+^ exchanger located on the cell surface and highly activated under acidic pH. It plays a critical role in maintaining cellular pH homeostasis by transporting one proton outside of a cell in exchange for moving an extracellular sodium ion inside the cell (33). Our data suggest that after TMEM175, NHE1 becomes another interesting proton efflux channel on lysosomes, but under a pathogenic condition (*e.g.*, atherosclerosis).

The 9p21.3 locus is the most complicated genetic locus. In addition to CAD and MI, the genomic variants at the 9p21.3 CAD locus showed significant association with many other diseases and genetic traits, including but not limited to various cancers, abdominal aortic aneurysm, peripheral artery disease, ischemic stroke, heart failure, coronary artery calcification, cerebral aneurysm, carotid plaque burden, diabetes, cataracts, glaucoma, migraine without aura, TC, and LDL-c (http://www.genome.ucsc.edu/ NHGRI-EBI Catalog of Published Genome-Wide Association Studies). It is important to note that the association between 9p21.3 variants and TC and LDL-c was weak because it was not identified in typical GWAS for lipids but instead showed up in a huge meta-analysis with more than 220 GWAS populations (60). We did not find any significant difference for TG, TC, HDL-c, or LDL-c between *ApoE*^*−/−*^*ANRIL* mice and control *ApoE*^*−/−*^ mice fed with a Western diet (Figs. 2 and S1). It is likely that the effect of the 9p21.3 locus on lipid levels is too weak to be detected in our mouse studies.

One limitation should be noted for the present study. Our transgenic overexpression mice target on *ANRIL*, which is a primate-specific gene, and does not exist in mice. However, transgenic ectopic overexpression mice were effective in exploring the *in vivo* functions of primate-specific genes, for example, *POP2*, *TMEM14B*, *ChAT*, *TOSPEAK*, and *MCHR1R2*, and uncovered novel mechanisms for inflammatory and innate response, neural progenitor proliferation and neurodevelopment, potential cholinergic vulnerability, severe speech impairment, and obesity, respectively (61, 62, 63, 64, 65). In addition, as the key molecules in the downstream signaling pathway for *ANRIL*-induced atherosclerosis, that is, *miR-181b-5p* and *TMEM106B*, are evolutionarily highly conserved between humans and mice, we believe that *ApoE*^*−/−*^*ANRIL* mice can serve as a model for chromosome 9p21.3-associated CAD and atherosclerosis despite the noted weakness. In fact, the sequences of *miR-181b-5p* are completely conserved in humans and mice (Fig. 4*C*). Moreover, the expression level of *ANRIL* in *ApoE*^*−/−*^*ANRIL* mice was comparable to that in human monocytes, the precursors of macrophages (Fig. 1*F*).

In summary, our study provides strong *in vivo* evidence to demonstrate the important causal role of *ANRIL* in atherosclerosis and show that increased lysosomal pH of macrophages is associated with atherosclerosis. We discover that the *ANRIL*–*miR-181b-5p*–TMEM106B-NHE1 axis acts as a novel molecular regulatory mechanism by which *ANRIL* promotes atherosclerosis. These results clarify a long-lasting issue in the field of CAD genetics, that is, the identity of the disease-causing gene at the most important genetic locus for CAD. Our results not only offer important new insights into the pathogenesis of CAD but also identify some novel targets for developing strategies to prevent and/or treat atherosclerosis. Moreover, our finding that NHE1 serves as an important new proton efflux channel involved in the homeostasis of lysosomal pH provides important insights into the biology of lysosomes and the role of lysosomal pH in atherosclerosis.

## Experimental procedures

### Study approval

Animal experiments were performed according to the guidelines from the Care and Use of Experimental Animals for Research by the Ministry of Science and Technology of the P. R. China. The animal experiments were approved by the Ethics Committee on Animal Research of Huazhong University of Science and Technology (2006–398; GB/T 35892-2018). The human studies were approved by the Ethics Committee of Huazhong University of Science and Technology (No. [2018]S353) and performed in compliance with the Declaration of Helsinki.

### Mice

A 3.8-kb human *ANRIL* complementary DNA (cDNA) fragment (NR_003529) was cloned into pcDNA3.1 to create the pcDNA3.1-*ANRIL* plasmid as reported (66). The pcDNA3.1-*ANRIL* plasmid was linearized with restriction digestion, purified with agarose gel electrophoresis, and injected into fertilized eggs derived from mouse strain C57BL/6N. The injected zygotes were then placed back into the female foster for developing *TgANRIL* mice. The F0 mice were bred with C57BL/6N mice to yield F1 heterozygous *TgANRIL* mice. Heterozygous *TgANRIL* mice were crossed with apolipoprotein E–deficient (*ApoE*^*−/−*^) mice on C57BL/6N background to develop *ApoE*^*−/−*^*ANRIL* mice. Mice were fed with a standard chow diet for 4 weeks and then with a Western diet (MD12015A, 21% fat, 0.5% cholesterol; Research Diets Inc) for 14 weeks to develop a mouse model for atherosclerosis.

### Human studies

Human arterial samples were collected from patients undergoing surgical resection of the aortic wall, and the non-CAD samples were from explanted hearts from cardiac transplantation. The tissue samples were all from medically needed procedures and would normally be discarded after the procedures.

### Plasmids

The cDNA for the human *TMEM106B* gene (NM_018374) and human *NHE1* gene (NM_003047.5) were amplified by PCR analysis using cDNA obtained by reverse transcription of mRNAs from HEK293 cells as a template and subcloned into the pEGFP-C1 vector and pCMV-3xFlag vector, resulting in respective mammalian expression plasmids GFP-TMEM106B, Flag-TMEM106B, GFP-NHE1, and Flag-NHE1. The expression plasmids for GST-fusion proteins GST-TMEM106B and GST-NHE1 were created by PCR amplification of *TMEM106B* and *NHE1* coding regions from GFP-TMEM106B and GFP-NHE1 and subcloned into the pGEX4T-1 vector, resulting in plasmids GST-TMEM106B and GST-NHE1. The 3′UTR of *TMEM106B* was PCR-amplified from human genomic DNA and subcloned into the pMIR-GLO vector, resulting in a luciferase reporter pMIR-TMEM106B-3′UTR.

For creating various truncation mutants of *TMEM106B* or *NHE1*, we used PCR analysis to amplify individual fragments of *TMEM106B* or *NHE1* using GFP-TMEM106B or GFP-NHE1, respectively, as the template and subcloned each fragment into the pEGFP-C1 vector. Point mutations were introduced into respective expression plasmids using the PCR-based method as previously reported (67).

Plasmids for GFP-tagged and RFP-tagged TMEM106B and NHE1 at either the N terminus or C terminus were constructed using standard molecular cloning protocols.

The microRNA mimics and inhibitors for miR181b-5p (*miR-181b-5p* mimics, *miR-181b-5p* inhibitor) or negative control miRNA (Ncontrol, NC inhibitor) were from GenePharma.

### Analyses of atherosclerosis and blood lipids

Atherosclerosis in mice was evaluated as previously described (68). Mice were anesthetized with an intraperitoneal injection of sodium pentobarbital (50 mg/kg body weight) and then euthanized by cervical dislocation. Aortas and aortic roots were excised and processed for histological analysis. Blood samples were collected for lipid analysis. The colorimetric enzymatic method was used to determine plasma TC and TG concentrations. The lipoprotein cholesterol substrate method was used to determine the levels of HDL-c and LDL-c. The left ventricle was injected with saline, and the adventitia fat was removed. The heart and the whole aorta were separated and fixed overnight in 4% paraformaldehyde. The heart was dehydrated overnight in 30% sucrose at 4 °C, embedded in OCT compound, and sectioned to obtain continuous frozen sections (8 μm) of aortic roots. Three sections per mouse were stained for lesion areas with oil red O. The aorta was cut open longitudinally, fixed on a black silicon bed, and stained with oil red O for 3 h at room temperature. The lesion areas were measured using ImageJ software and quantified.

### Cell culture

Primary human coronary artery endothelial cells (MeisenCTCC) were cultured in endothelial cell growth basal medium with endothelial cell growth factor added, and primary human coronary artery smooth muscle cells (MeisenCTCC) were cultured in smooth muscle cell growth basal medium with 10% fetal bovine serum and 1% antibiotics at 37 °C and 5% CO_2_.

### Isolation of primary macrophages

Mouse primary macrophages were isolated using bone marrow as described (69, 70). Bone marrow was flushed from mouse femurs and tibias, filtered, centrifuged, and mixed with red cell lysis buffer. The cells were centrifuged and plated in RPMI1640 (CM10041, Macgene) supplemented with 10% fetal bovine serum (10100147, Gibco), 2% 4-(2-hydroxyethyl) piperazine-1-ethanesulfonic acid (Hepes) (BP-299-100, Thermo Fisher Scientific), 1% nonessential amino acids (M7145, Sigma), 1% antibiotic-antimycotic solution (15240-096, Life Technologies), and 10 ng/ml macrophage colony-stimulating factor (416-ML, R&D Systems). Human primary macrophages were obtained by adding 20 ng/ml of macrophage colony-stimulating factor for 7 days to the isolated PBMC, which were isolated as previously described (71).

### Cell transfection

Overexpression of *ANRIL* in human macrophages utilized the lentiviral system. Macrophages were infected with lentiviruses for 72 h in the antibiotic-free medium. The cells were then washed and incubated in a fresh medium for an additional 48 h before being used for WB or immunostaining. The TransIT-X2 Dynamic Delivery System was used for transfection of plasmids and siRNA. The plasmid or siRNA was mixed with the transfection reagent for 15 min and then added into the cells for an additional 48h.

### Lysosomal pH measurement

Lysosensor green DND-189 (A66436, Invitrogen) was used to assess the acidity of lysosomes, and both showed staining signals only for low lysosomal pH (72). Primary macrophages were incubated with Lysosensor (1 μM) for 2 h at 37 °C, fixed, and imaged under a FV3000 confocal microscope. Mean florescence intensity was quantitated.

We also measured lysosomal pH using a method with a combination of pH-insensitive and pH-sensitive dyes as described earlier (59). Cells were cultured overnight in the presence of Fluorescein-tetramethylrhodamine dextran (D1951, Invitrogen), washed, and chased in regular growth media for 2 h. The cells were fixed with 4% paraformaldehyde and balanced in pH calibrated standard solutions (pH 4, 5, 6, and 7) for 10 min. All cells were photographed using the same microscope. The fitting of FITC/TRITC ratios to the calibration group was used to calculate the lysosomal pH of the experimental group.

### Lysosomal fractionation

Lysosomal fractions were isolated from cultured cells by density gradient separation (34,200 rpm, 2 h) using the lysosome enrichment kit for tissue and cultured cells and protocols from Pierce (89839, Thermo Fisher Scientific) (73).

### Luciferase assays

For luciferase assays, HEK293 cells were plated in a 24-well plate for 24 h and transfected with plasmid and microRNA. Transfected cells were used for luciferase assays 48 h after transfection using the dual-luciferase reporter assays (Promega) according to the manufacturer's instructions as described (74, 75, 76).

### Protein analyses

Protein–protein interaction analysis was carried out using GST pull-down assays and Co-IP assays as previously described (77, 78). Western blot analysis was performed as described (77, 78). In brief, cultured cells and minced mouse tissue samples were lysed in Western and IP cell lysis buffer (20 mM Tris, pH 7.5, 150 mM NaCl, 1 mM EDTA, 1% Triton X-100, proteinase inhibitor cocktail, and phosphatase inhibitor cocktail). Protein samples were mixed with 1x SDS-loading buffer, boiled for 15 min, and analyzed with SDS-PAGE electrophoresis and Western blotting.

Co-immunostaining of TMEM106B and NHE1 was carried out as described previously (79).

### RT-qPCR analysis

RT-qPCR analysis was carried out as described (80, 81, 82). Briefly, total RNA was isolated from cultured cells or minced mouse tissue samples with the RNAiso Plus (Takara Biotech) and reversely transcribed to cDNA by the RT Reagent Kit (Promega). Quantitative PCR analysis was performed using the Hieff qPCR SYBR Green Mix (Yeasen) on a QuantStudio 12K Flex Real-time PCR System (Applied Biosystems). The sequences of the RT-qPCR primers can be seen in Table S1.

### Statistical analysis

GraphPad Prism 8 was used for statistical analysis. The data were expressed as mean ± SD. The comparison between two groups was made by unpaired two-tailed *t*-tests (normal distribution) or nonparametric tests (non-normal distribution). To compare the means of more than two groups, we used multiple *t*-tests and one-way ANOVA tests with Bonferroni adjustment for multiple testing. Two-way ANOVA with Bonferroni adjustment was used when there were more than two independent variables. *p* < 0.05 was considered significant.

## Data availability

The authors declare that all supporting data are available within the article and the Supporting Information. We predicted protein–protein interactions using an old version of the BioGRID database (version 4.3.196). Data can be downloaded from the website (https://downloads.thebiogrid.org/BioGRID/Release-Archive/BIOGRID-4.3.196/).

## Supporting information

This article contains supporting information.

## Conflict of interest

The authors declare that they have no conflicts of interest with the contents of this article.
