# Supplementary material for: LncRNA-induced lysosomal localization of NHE1 promotes increased lysosomal pH in macrophages leading to atherosclerosis
Source: J Biol Chem. 2025 May 16;301(6):110246. doi: 10.1016/j.jbc.2025.110246 (PMC12192704; doi:10.1016/j.jbc.2025.110246)
Supplement: Supporting information [file mmc1.docx]

**Supporting Information**

**LncRNA-induced lysosomal localization of NHE1 promotes increased lysosomal pH in macrophages leading to atherosclerosis**

Pengcheng Shi^1,‡^, Bo Tang^1,‡^, Wen Xie^1,‡^, Ke Li^1,‡^, Di Guo^1^, Yining Li^1^, Yufeng Yao^1^, Xiang Cheng^2^, Chengqi Xu^1^, and Qing K. Wang^1,3,*^

From the ^1^Center for Human Genome Research, Key Laboratory of Molecular Biophysics of the Ministry of Education, College of Life Science and Technology; ^2^Department of Cardiology, Union Hospital, Tongji Medical College; ^3^Maternal and Child Health Hospital of Hubei Province, Women and Children’s Hospital of Hubei Province, Huazhong University of Science and Technology, Wuhan, P. R. China

^‡^These authors contributed equally to this work.

^*^Correspondence to: Qing K. Wang, Center for Human Genome Research and College of Life Science and Technology, Huazhong University of Science and Technology, 1037 Luoyu Road, Wuhan, P. R. China. Email: [qingwang118@qq.com](mailto:qingwang118@qq.com)

**Supplemental Figures**


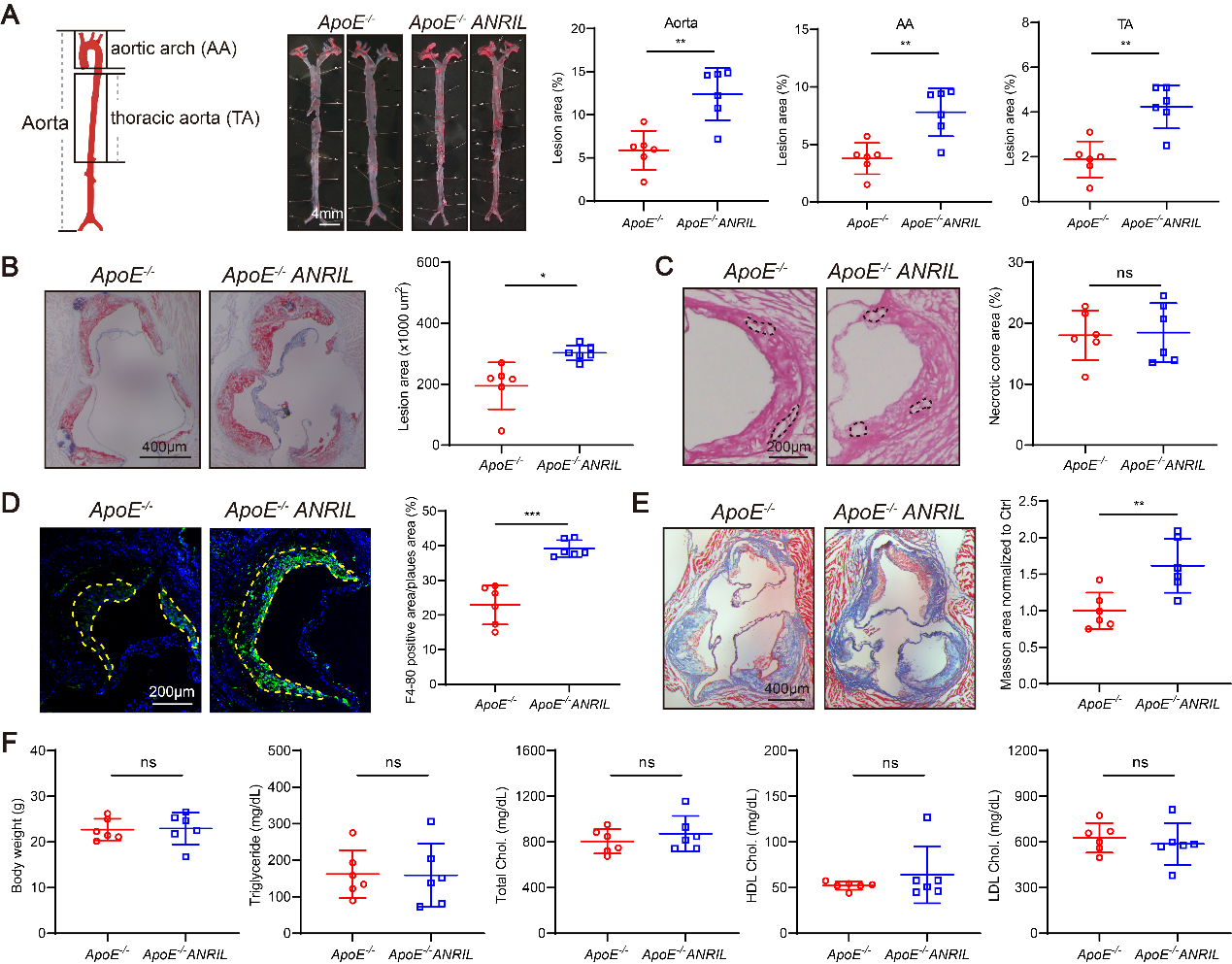


**Figure S1. Female *ApoE^-/-^ANRIL* mice show aggravated atherosclerosis.** *A*, female mice were fed with a standard chow diet for 4 weeks and then with a WD for 14 weeks. Representative lipid/oil red O-stained enface images of aortas. n = 6. *B*, representative lipid/oil red O-staining of aortic root sections. n = 6. *C*, representative necrotic core areas of plaques in aortic root sections. n = 6. *D*, representative anti-F4-80 immunofluorescent images of aortic root sections. n = 6. *E*, representative Masson-staining of aortic root sections. n = 6. *F*, body weight, triglyceride, total cholesterol, HDL cholesterol, and LDL cholesterol levels. n = 6. **P*<0.05, ***P*<0.01, ****P*<0.001, ns, not significant. A, B, C, D, E, and F, unpaired two-tailed *t*-test; F (HDL), nonparametric test.


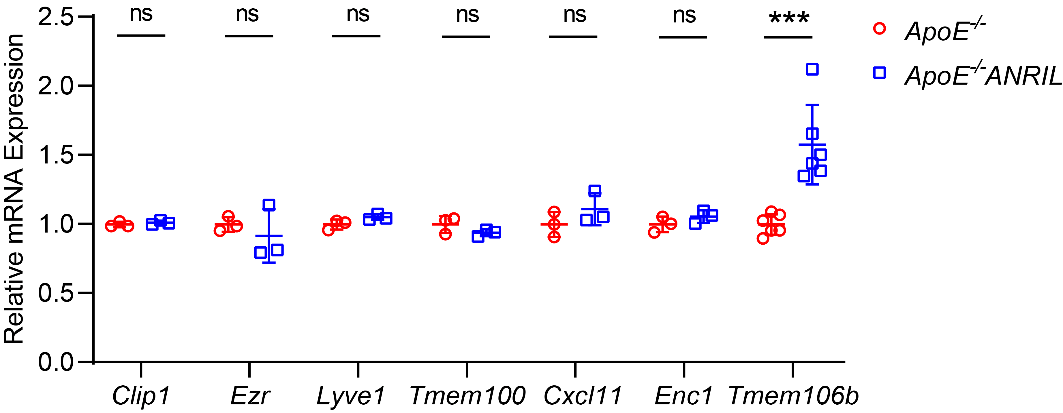


**Figure S2. Expression of *Tmem106b* was regulated by *ANRIL* overexpression.** RT-qPCR analysis to detect expression of *Clip1*, *Ezr*, *Lyve1*, *Tmem100*, *Cxcl11*, *Enc1*, and *Tmem106b* in atherosclerotic lesions from *ApoE^-/-^ANRIL* mice. n = 3. ****P*<0.001, ns, not significant. Multiple *t*-test.


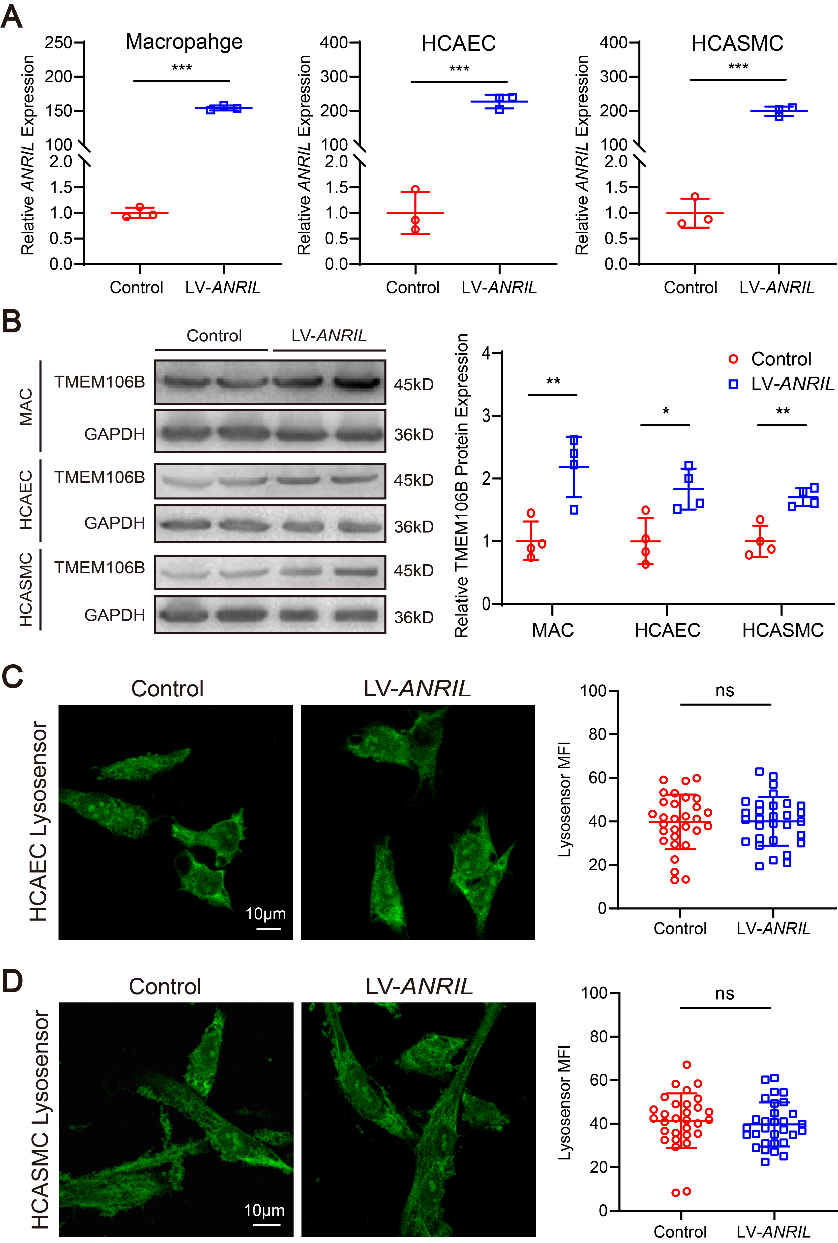


**Figure S3. *ANRIL* overexpression did not affect lysosomal pH in primary HCAECs or HCASMCs.** *A*, RT-qPCR analysis for *ANRIL* in macrophages, HCAECs, and HCASMCs with LV-*ANRIL* treatment. n = 3. *B*, western blot analysis for TMEM106B in macrophages, HCAECs, and HCASMCs with *ANRIL* overexpression. n = 4. *C*, human primary HCAECs with *ANRIL* overexpression stained for Lysosensor. n = 30. *D*, human primary HCASMCs with *ANRIL* overexpression stained for Lysosensor. n = 30. **P*<0.05, ***P*<0.01, ****P*<0.001, ns, not significant. A, C, and D, unpaired two-tailed *t*-test; B, multiple *t*-test.


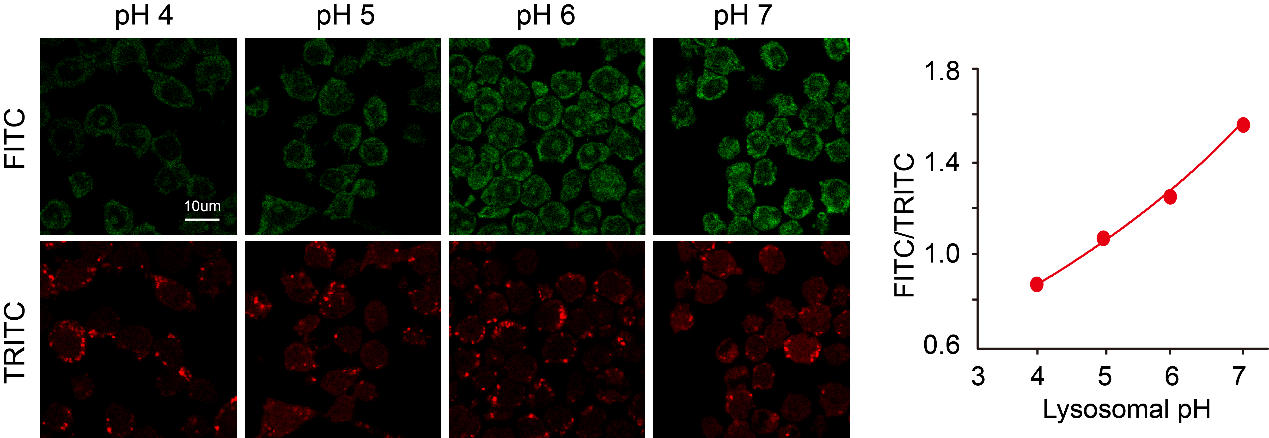


**Figure S4. Lysosomal pH measurements with a combination of pH-insensitive TRITC dye and pH-sensitive FITC dye.** Macrophages were incubated with dextran conjugated with pH-sensitive FITC fluorescein (green) and pH-insensitive tetramethylrhodamine (red). FITC and TRITC intensities per cell were quantified and the ratio of FITC/TRITC was calculated. Lysosomal pH was determined from the calibration curve generated from the FTIC/TRITC value of permeabilized cells in various calibration standard solutions with different pH values.


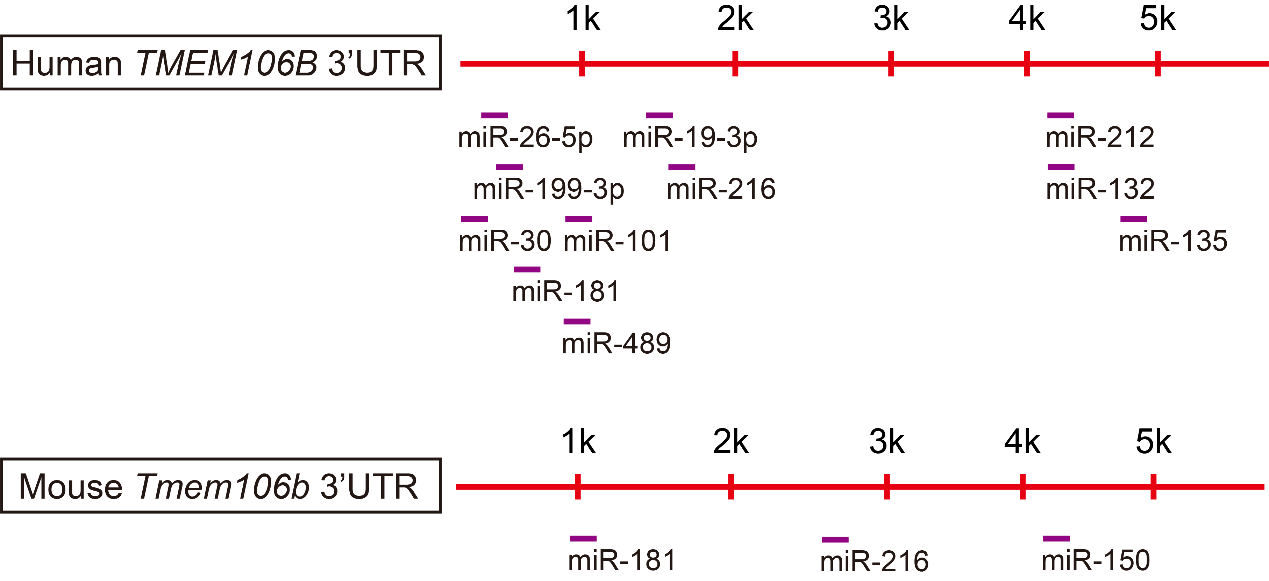


**Figure S5. Targetscan predicts miRNAs that bind to human and mouse *TMEM106B* 3’UTR.** Targetscan identified 11 microRNAs at the 3’UTR of human *TMEM106B* mRNA and 3 microRNAs at the 3’UTR of mouse *Tmem106b* mRNA.


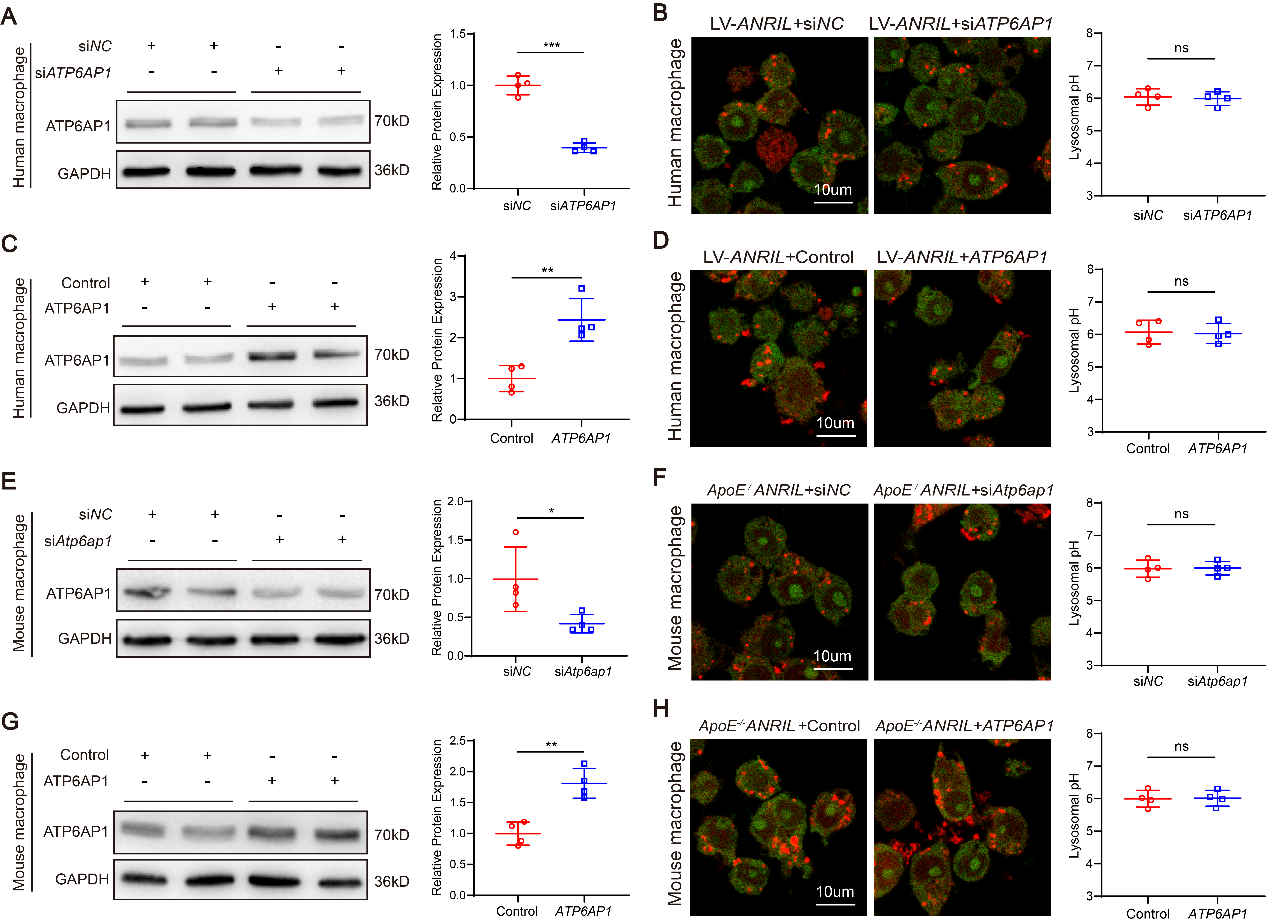


**Figure S6. *ATP6AP1* does not reverse the increased lysosomal pH caused by *ANRIL* overexpression.** *A* and *C*, ATP6AP1 was successfully knocked down and overexpressed in human macrophages. n = 4. *B* and *D*, lysosomal pH measurements for human PBMC-induced macrophages with *ANRIL* overexpression (LV-*ANRIL*) were transfected with si*ATP6AP1* vs. si*NC*, or *ATP6AP1* expression plasmid vs. Control plasmid. n = 4 samples with >50 cells were counted in each sample. *E* and *G*, ATP6AP1 was successfully knocked down and overexpressed in mouse macrophages. n = 4. *F* and *H*, lysosomal pH measurements for *ApoE^-/-^ANRIL* macrophages transfected with si*ATP6AP1* vs. si*NC*, or *ATP6AP1* expression plasmid vs. Control plasmid. n = 4 samples with >50 cells counted in each sample. **P*<0.05, ***P*<0.01, ****P*<0.001, ns, not significant. Unpaired two-tailed *t*-test.


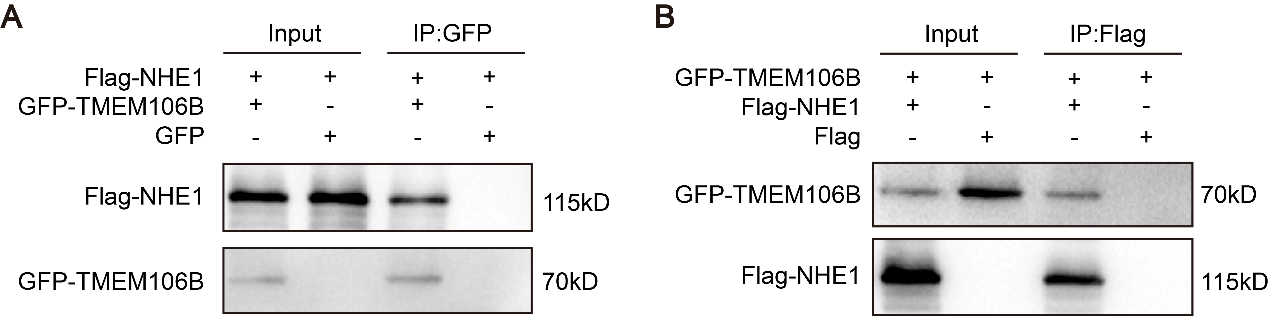


**Figure S7. TMEM106B interacts with NHE1.** *A*, Flag-NHE1 was co-transfected with the GFP vector or GFP-TMEM106B construct into HEK293 cells. Cell lysates were immunoprecipitated with anti-GFP, and western blot analysis was done with anti-Flag. *B*, GFP-TMEM106B was co-transfected with the Flag vector or Flag-NHE1 construct into HEK293 cells. Immunoprecipitation was done with anti-Flag, and western blot analysis was done with anti-GFP.


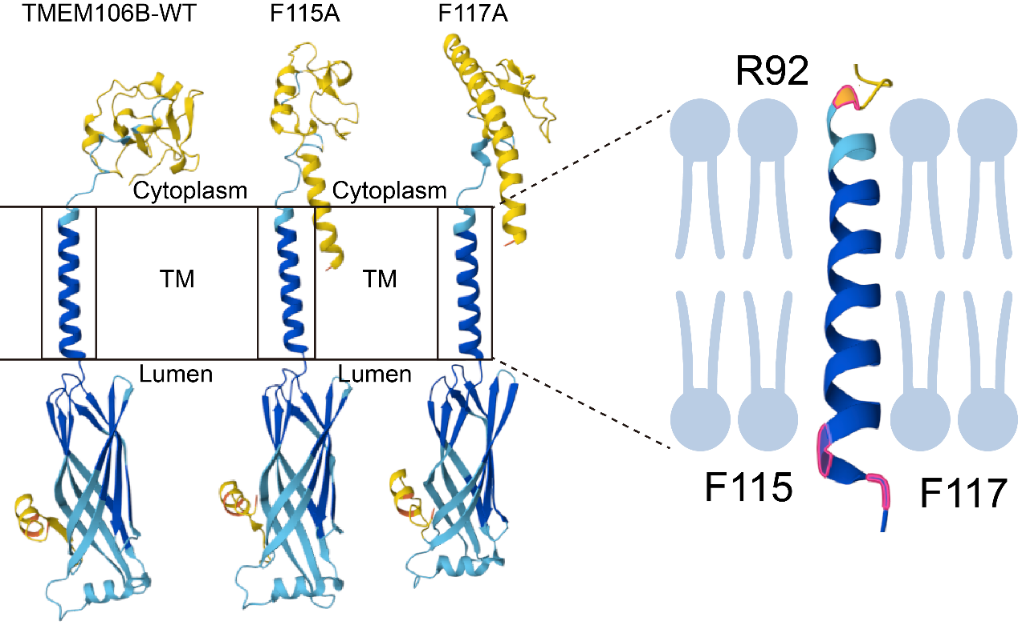


**Figure S8. Predicted structure of TMEM106B on lysosomal membranes by Chai Discovery.**

**Table S1. The sequences of the RT-qPCR primers.**

| Gene | Forward primer sequence 5’→3’ | Reverse primer sequence 5’→3’ |
| --- | --- | --- |
| *ANRIL-NR* | AACCTGAGCAGCTGGGACTA | TGTGTCCATAGCACCTTCCA |
| *ANRIL-DQ* | CCACATCCCTTGGAGTAATGA | CCTTTTATCACCCAGCTTCG |
| *CircANRIL* | GCTGGGATTACAGGTGTGAGACAC | GAATCAGAATGAGGCTTATTCTTCTC |
| *TMEM106B* | CCTACTTGTCAGGGAACAGGAAG | AACACAGCCAATCCAGAAAGGAG |
| *GAPDH* | ACATCGCTCAGACACCATG | TGTAGTTGAGGTCAATGAAGGG |
| *Il1b* | GAAATGCCACCTTTTGACAGTG | TGGATGCTCTCATCAGGACAG |
| *Tnfa* | CCCACGTCGTAGCAAACCA | ACAAGGTACAACCCATCGGC |
| *Il6* | TCGTGGAAATGAGAAAAGAGTTG | TCTGAAGGACTCTGGCTTTGTC |
| *Il10* | GGTTGCCAAGCCTTATCGGA | CACCTTGGTCTTGGAGCTTATT |
| *Tmem106b* | AACATTGGCCCACTTGATATGAA | GAGTGTCCAAAGTATGCTGTTGT |
| *Clip1* | ATGAGTATGCTAAAACCCAGCG | TCTCCAACTCGAAAGTCATCCA |
| *Ezr* | CCCAAAGATCGGCTTCCCTT | GTTTCCAACTGCTGTCGCTC |
| *Lyve1* | CCACAACTCATCCGACACCT | GCTGCTCCACTTGCAACAAA |
| *Tmem100* | GTGTACGGTCCCTAGCATGG | GTCAGGATGCAAGCTCACAG |
| *Cxcl11* | TGTAATTTACCCGAGTAACGGC | CACCTTTGTCGTTTATGAGCCTT |
| *Enc1* | GCAGATCCGGAGACCGAG | ACTGACATTTTGTTTCAACTCAGTA |
| *Gapdh* | AATGGTGAAGGTCGGTGTG | CTGGAAGATGGTGATGGGC |

**Table S2. The sequences of siRNA.**

| siRNA | sequence |
| --- | --- |
| Human si*TMEM106B* | CCAUUAUUGGUCCACUUGAUA |
| Mouse si*Tmem106b* | CUUGAUAUGAAGCAGAUUGAU |
| Human si*NHE1* | CGGUGAGCAGAUCAACAACAU |
| Mouse si*Nhe1* | GUAUGUGAAGAAGUGCCUGAU |
| Human si*ATP6AP1* | GCAUUGAGGAUUUCACAGCAU |
| Mouse si*Atp6ap1* | GCCCAUUUCAAUGUUUCCCAA |
